# Supplementary figures and images for: Imaging performance of portable and conventional ultrasound imaging technologies for ophthalmic applications
Source: PLoS One. 2024 May 13;19(5):e0300451. doi: 10.1371/journal.pone.0300451 (PMC11090327; doi:10.1371/journal.pone.0300451)

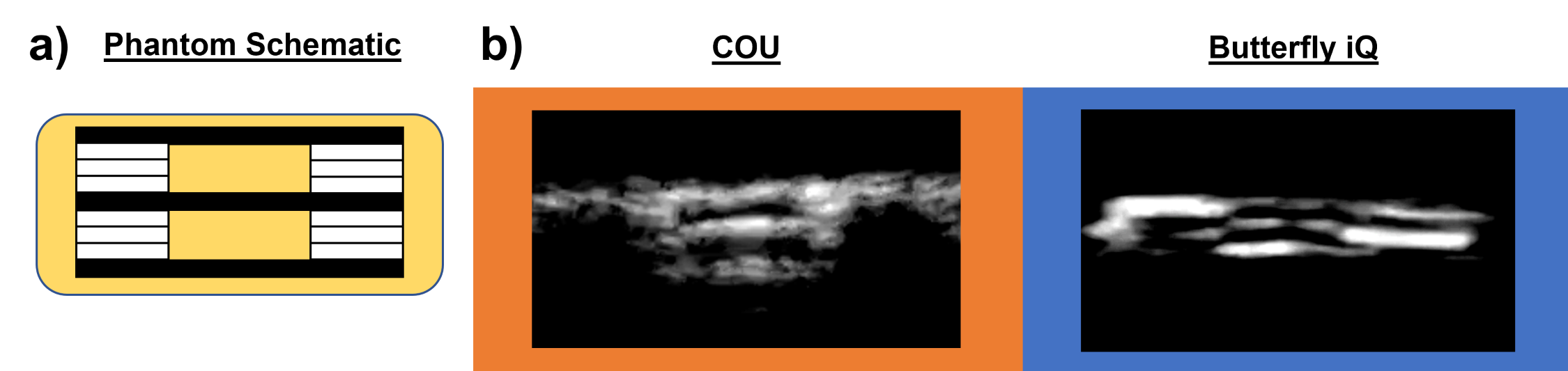

Supplement: S1 Fig — a) Diagram of model with three papers (black) each separated by three layers of tape (white) and gelatin (yellow). b) COU imaging is outlined in orange and Butterfly iQ imaging is outlined in blue. (TIF) [file pone.0300451.s001.tif]
