# Supplementary material for: Imaging performance of portable and conventional ultrasound imaging technologies for ophthalmic applications
Source: PLoS One. 2024 May 13;19(5):e0300451. doi: 10.1371/journal.pone.0300451 (PMC11090327; doi:10.1371/journal.pone.0300451)
Supplement: S1 File — (PDF) [file pone.0300451.s005.pdf]

Ultrasound Imaging Modality Evaluation

Scan QR Code to Start Questionnaire →

OR go to

https://tinyurl.com/y3pjs8gl

Browne Lab – Ermin Dzihic, David Horton, Josiah To, MD, MASc, Anderson Vu, MD, Andrew Browne, MD, PhD

1A

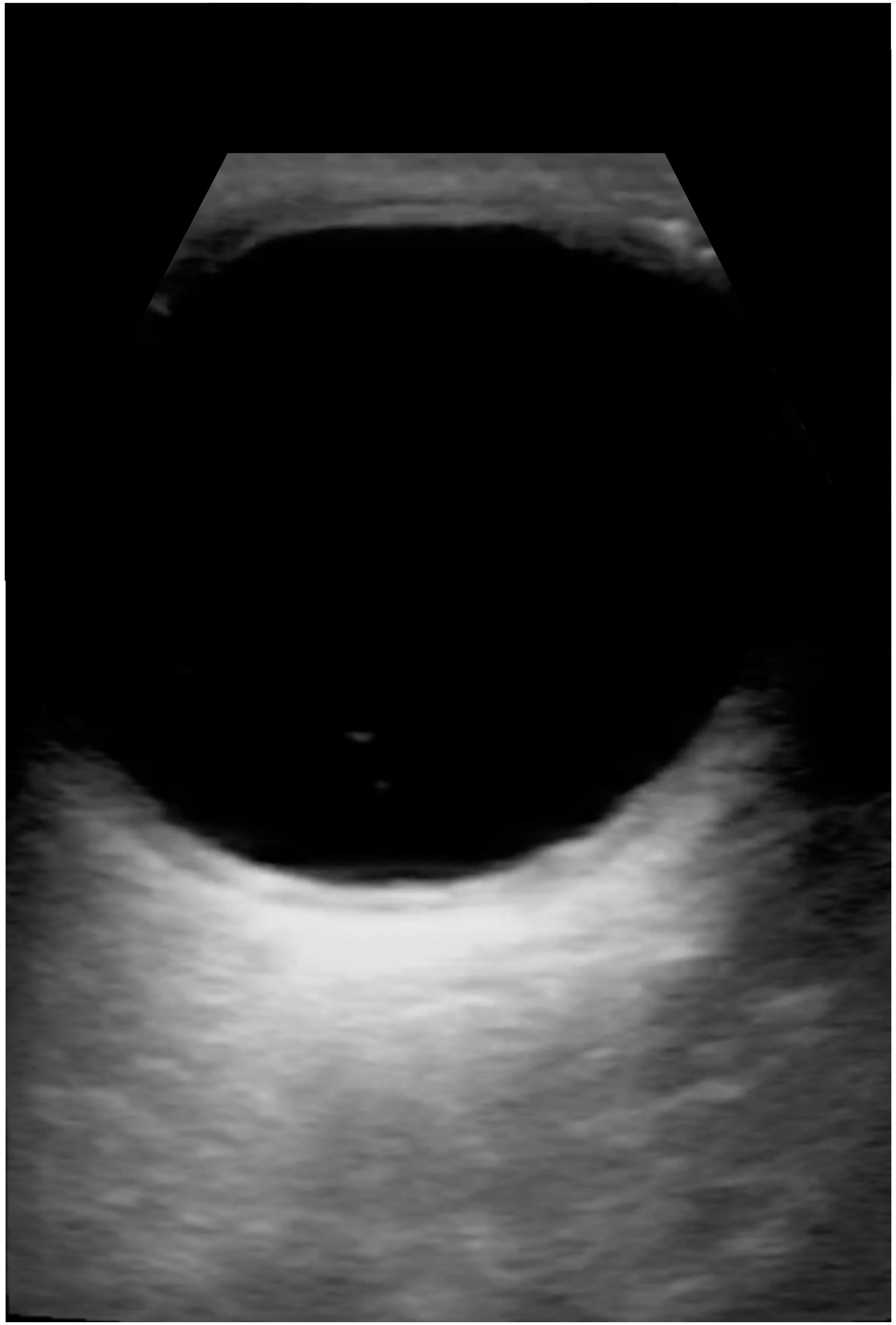

CC: Vitreous Hemorrhage
51 y/o m, hx DPR, c/o heme OD

1B

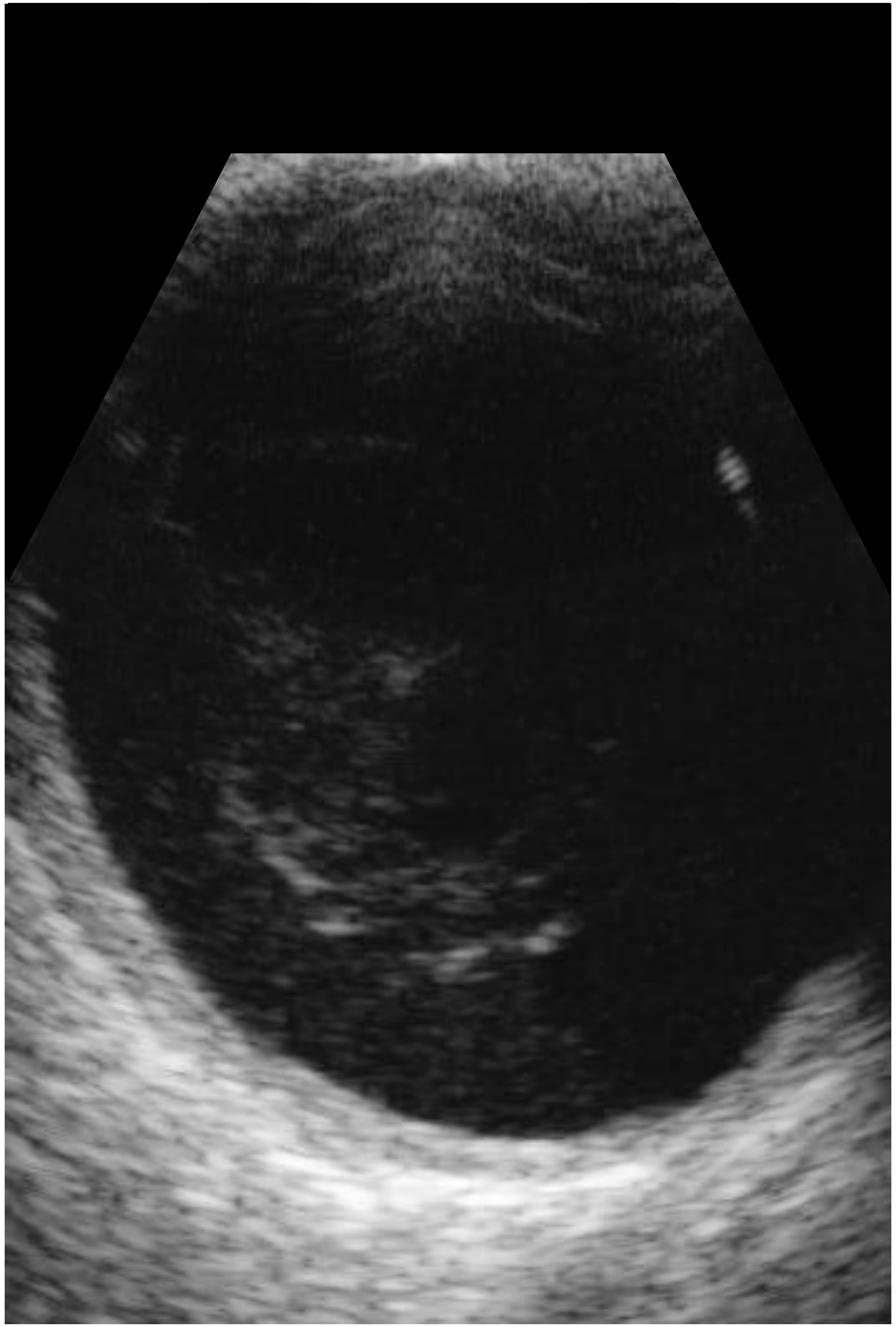

CC: Vitreous Hemorrhage
51 y/o m, hx DPR, c/o heme OD

1C

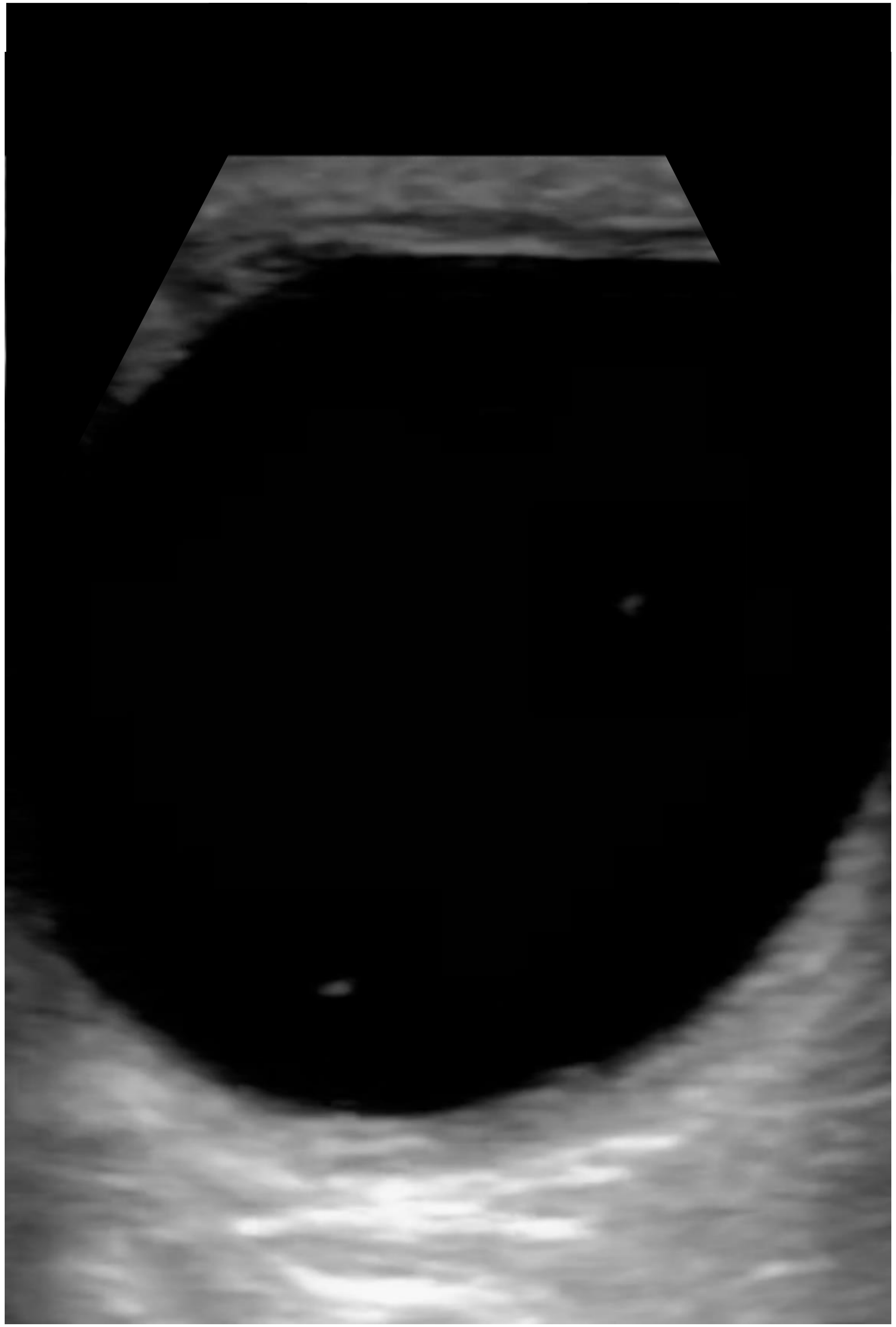

CC: Vitreous Hemorrhage
51 y/o m, hx DPR, c/o heme OD

1D

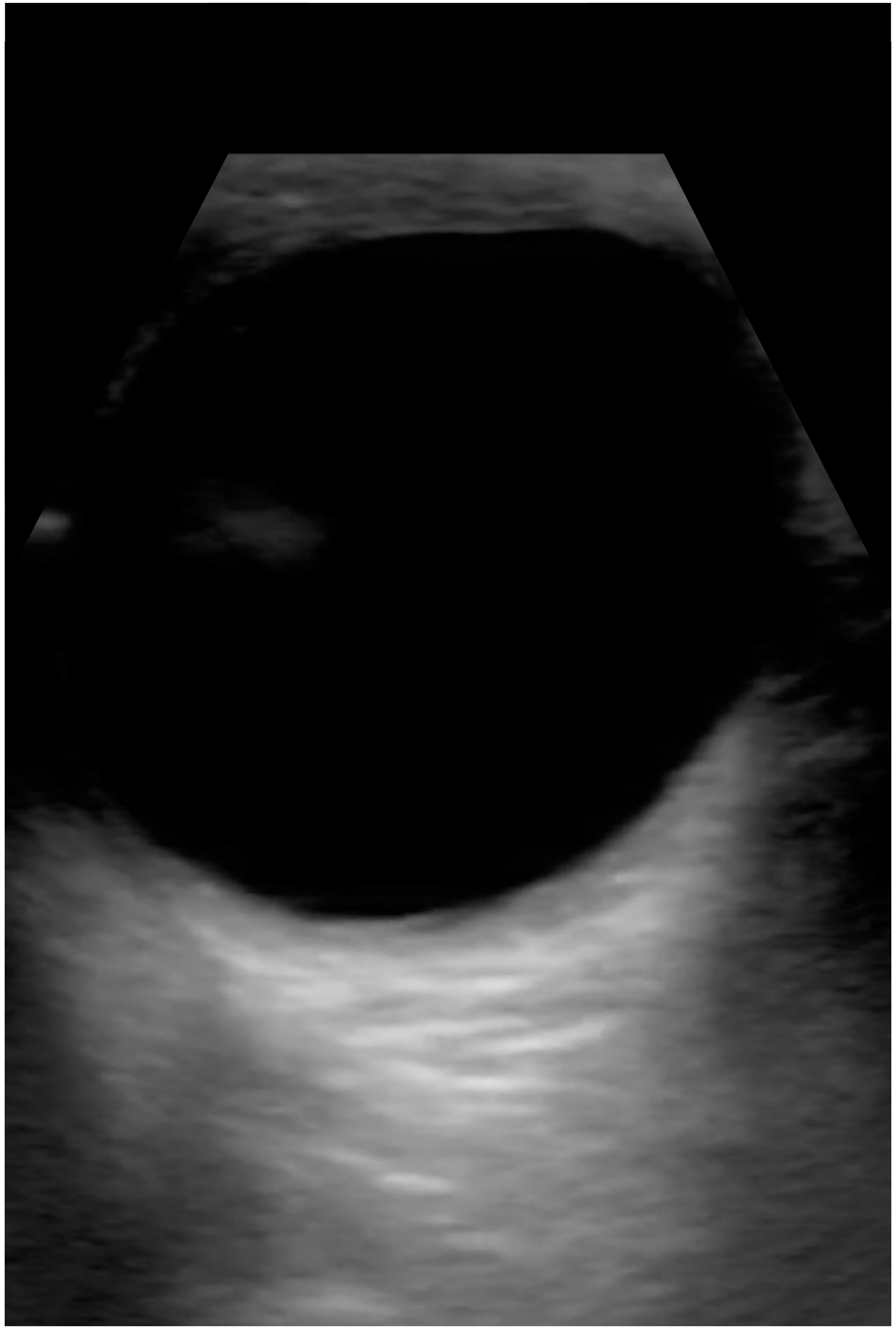

CC: Vitreous Hemorrhage
51 y/o m, hx DPR, c/o heme OD

1E

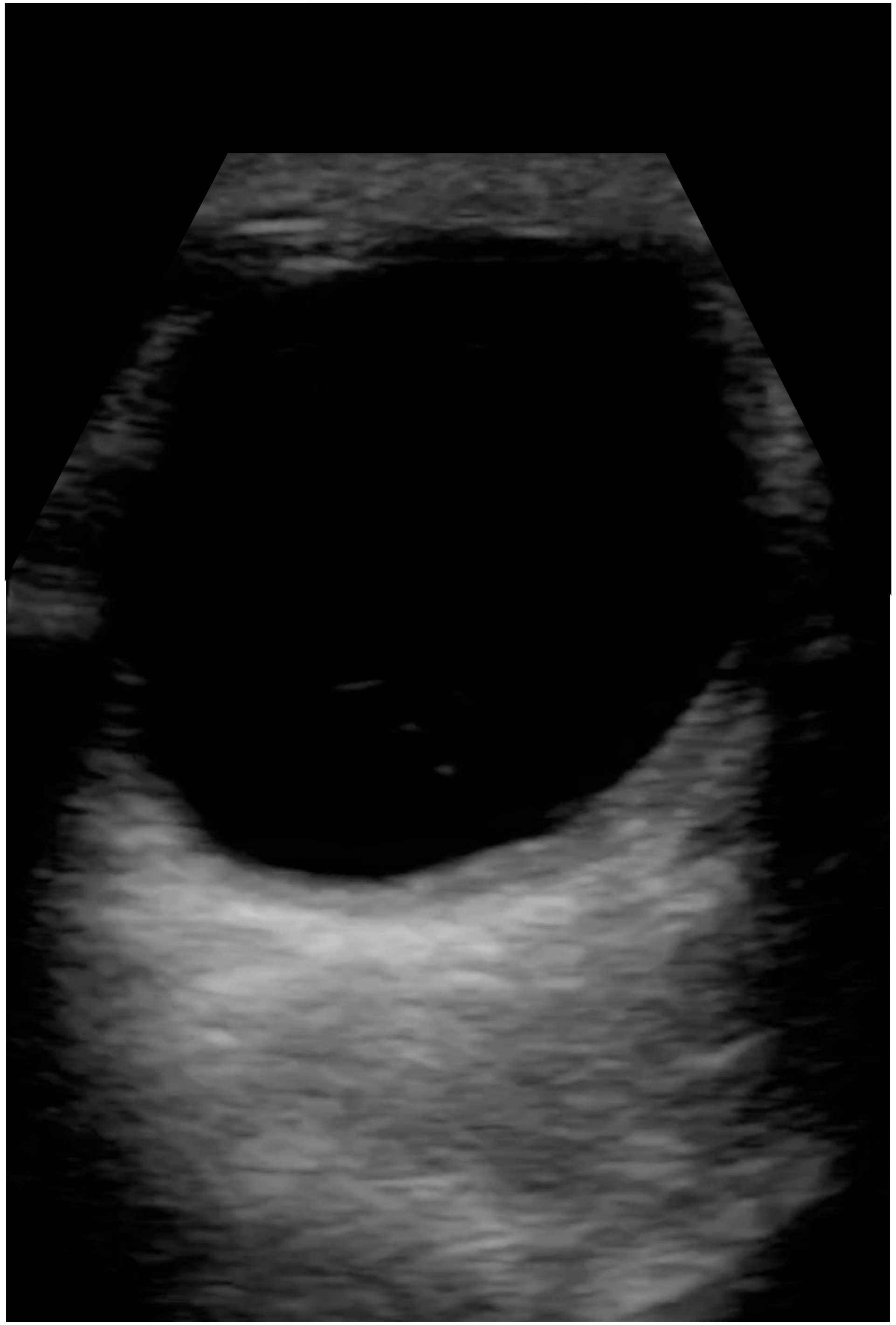

CC: Vitreous Hemorrhage
51 y/o m, hx DPR, c/o heme OD

2A

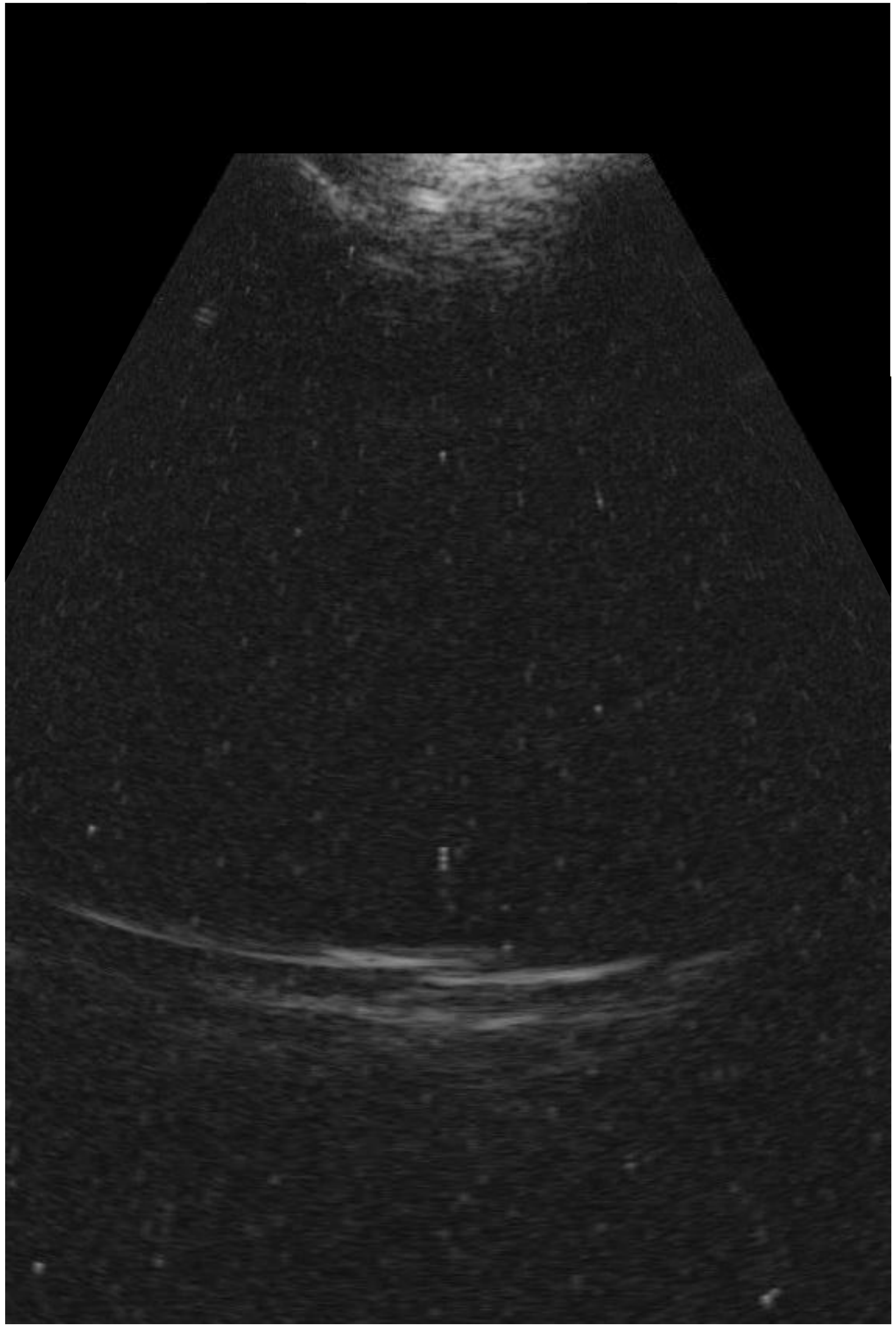

CC: Silicone Oil F/u
21 y/o M tractional RD OS s/p PPV/SOR/ phacoemulsification/ \anterior membrane dissection/SO

2B

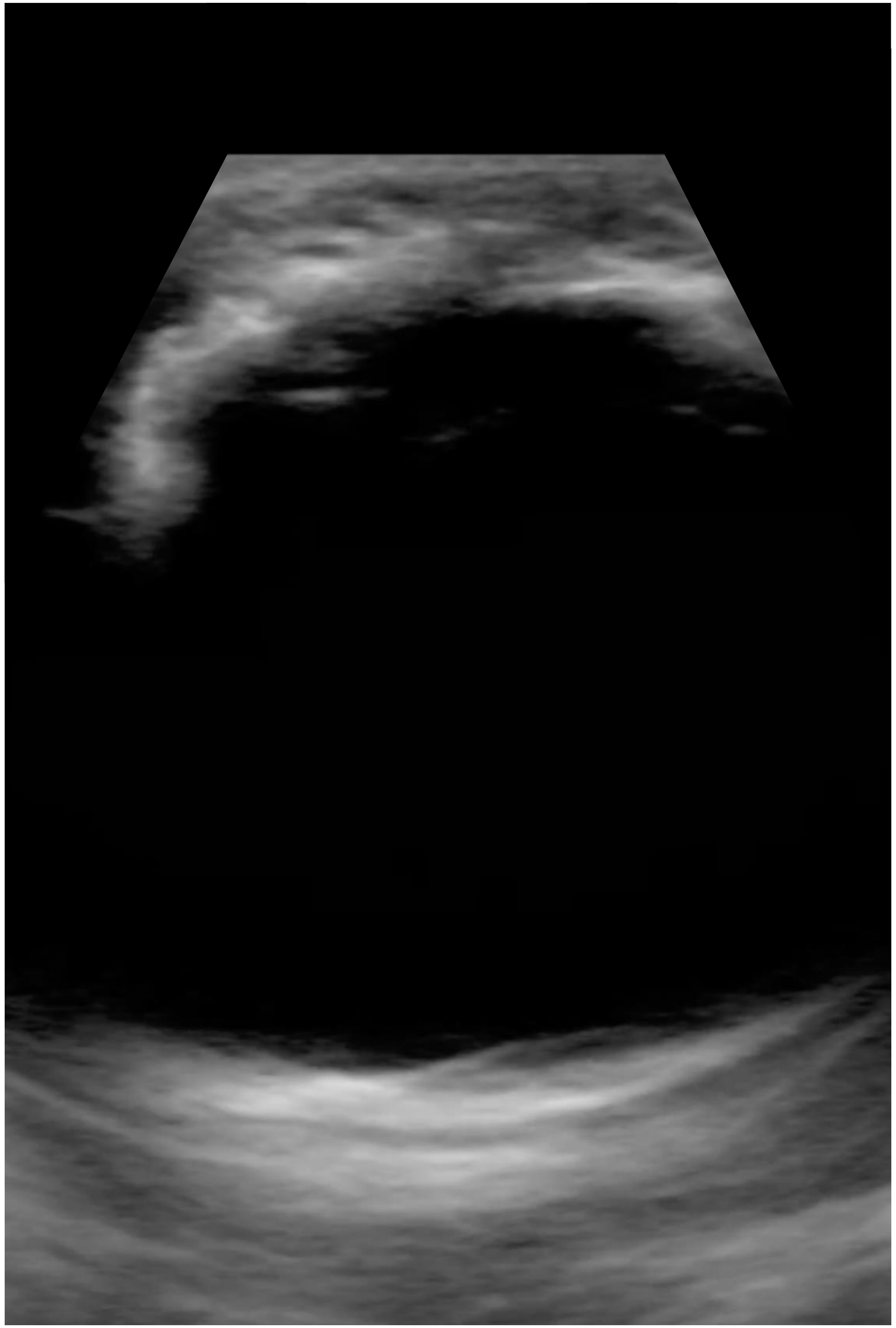

CC: Silicone Oil F/u
21 y/o M tractional RD OS s/p PPV/SOR/ phacoemulsification/ \anterior membrane dissection/SO

2C

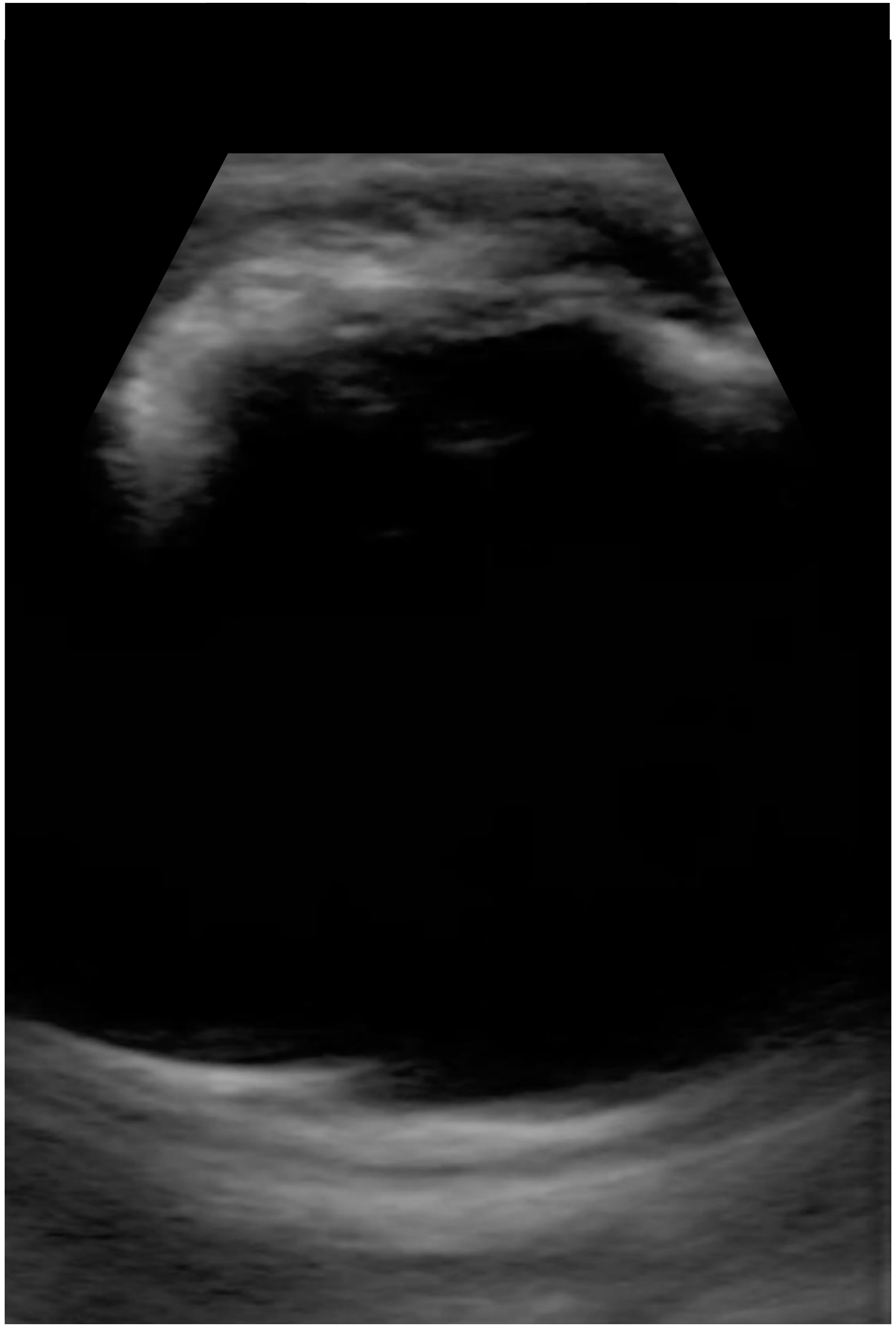

CC: Silicone Oil F/u
21 y/o M tractional RD OS s/p PPV/SOR/ phacoemulsification/ \anterior membrane dissection/SO

2D

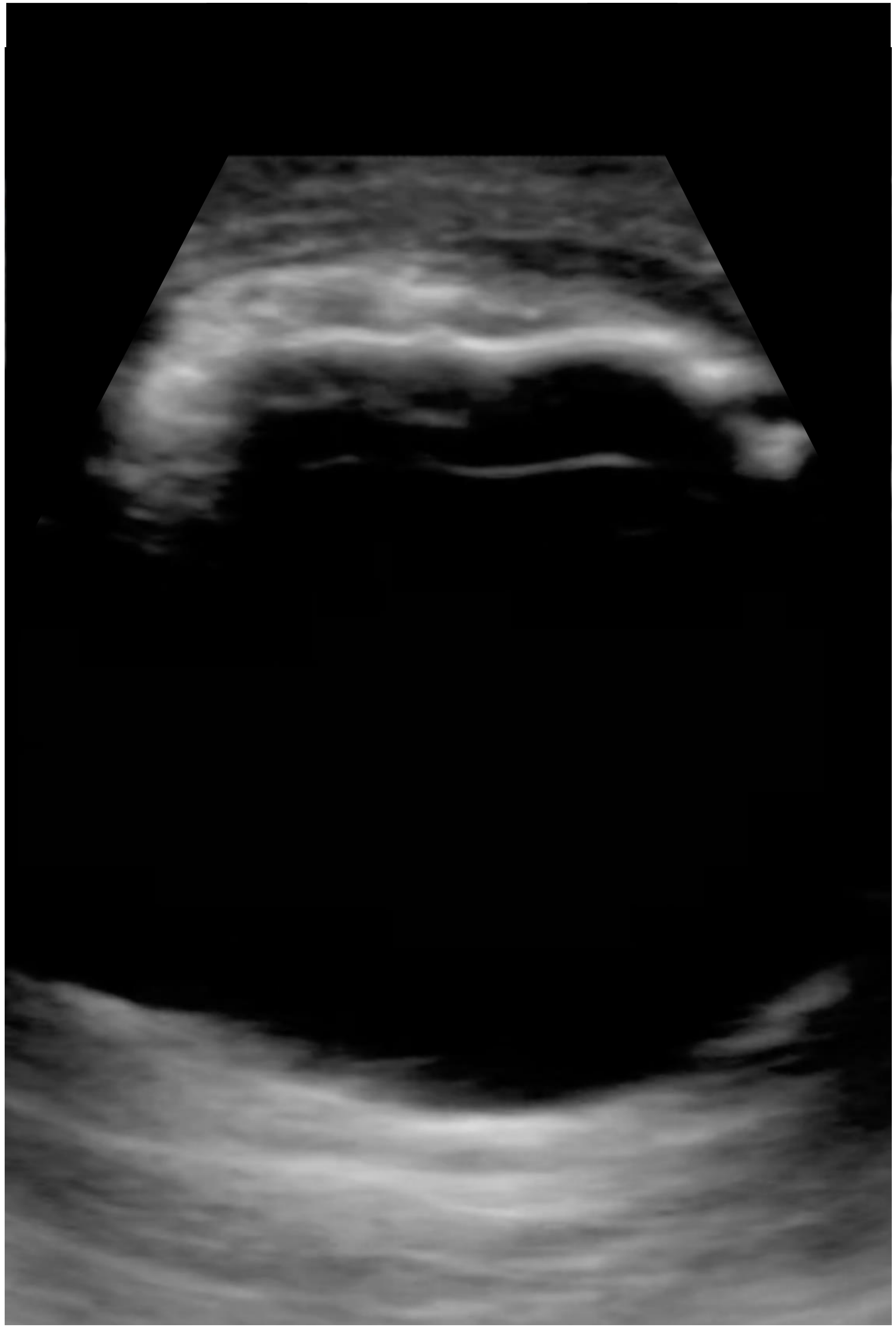

CC: Silicone Oil F/u
21 y/o M tractional RD OS s/p PPV/SOR/ phacoemulsification/ \anterior membrane dissection/SO

2E

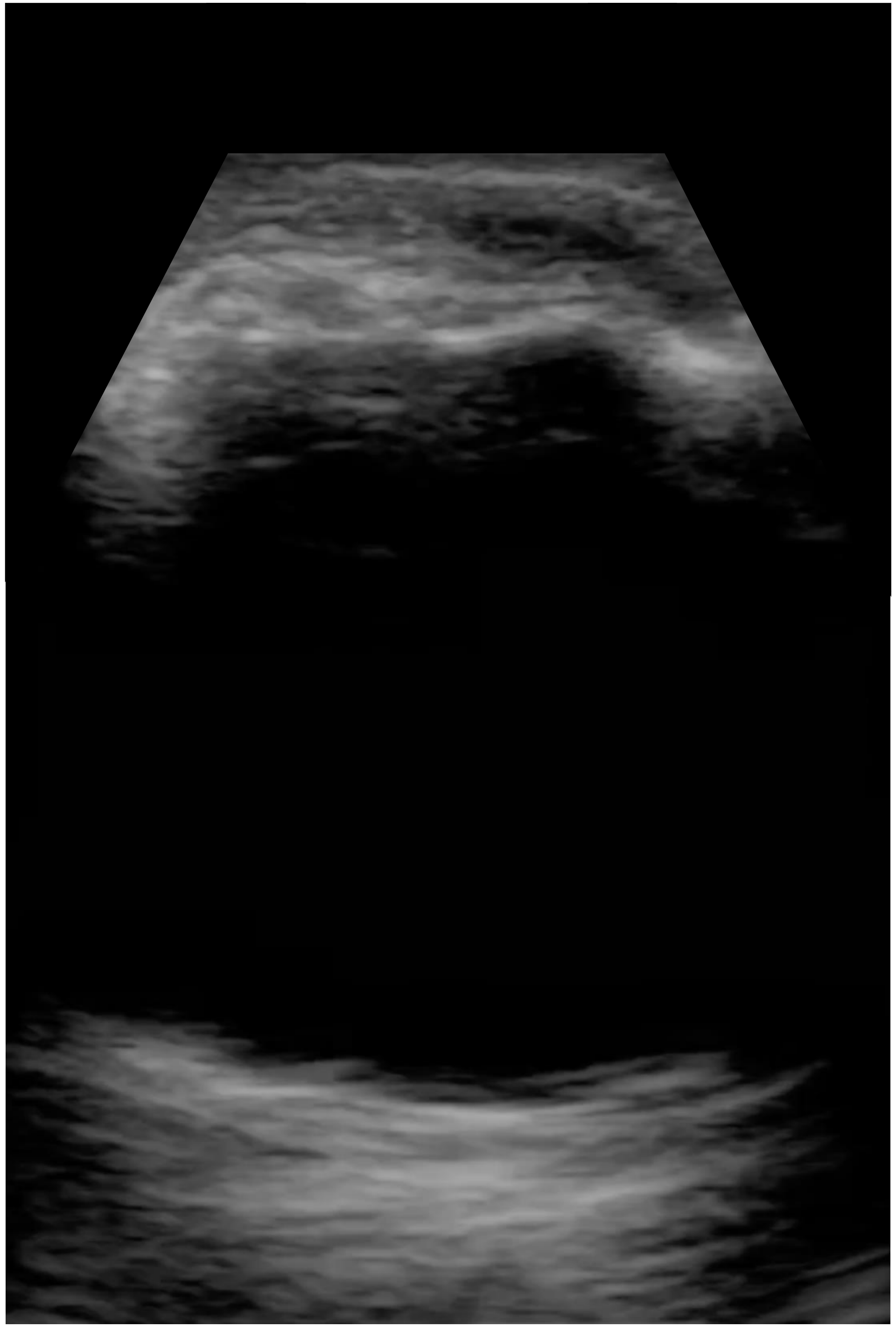

CC: Silicone Oil F/u
21 y/o M tractional RD OS s/p PPV/SOR/ phacoemulsification/ \anterior membrane dissection/SO

3A

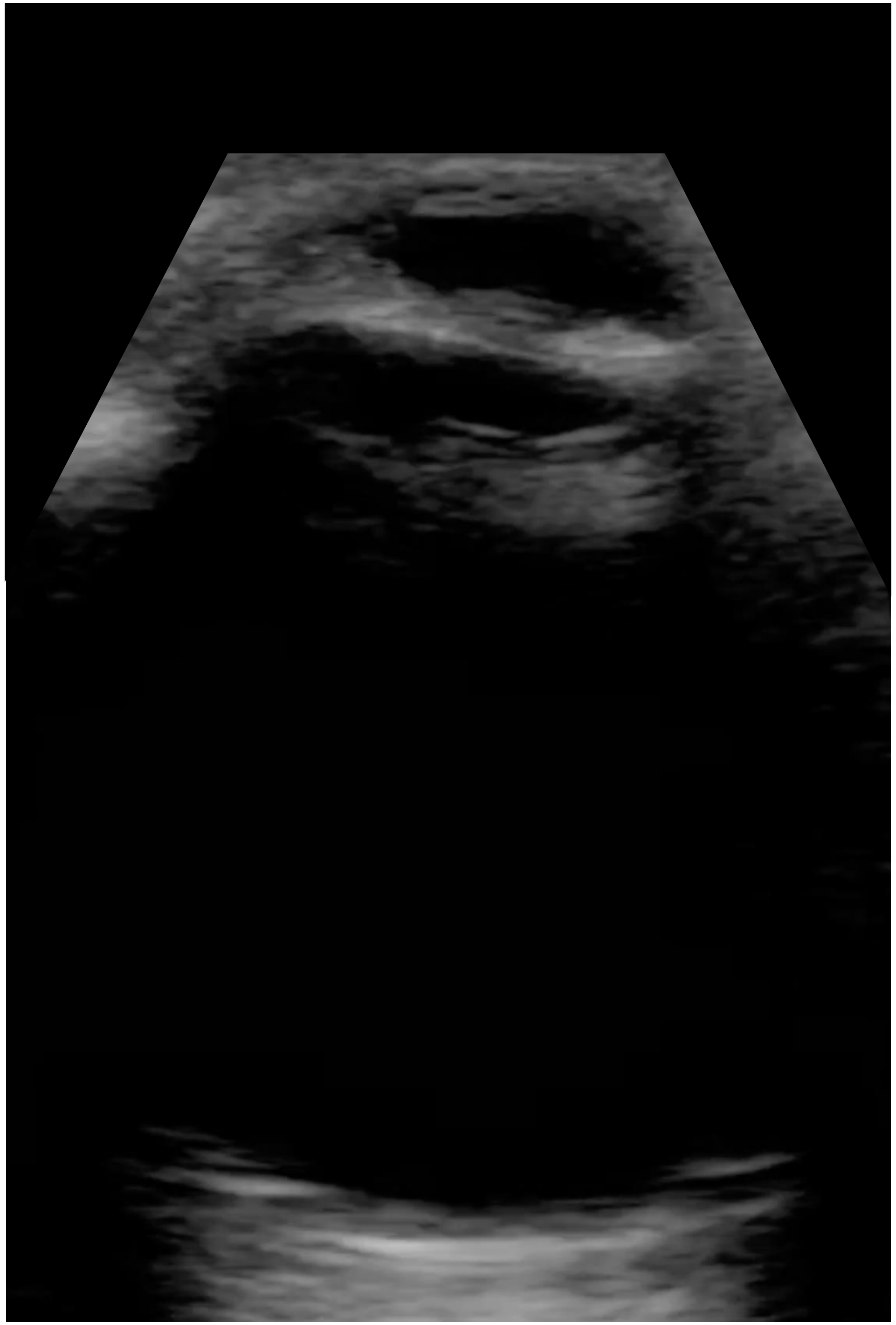

CC: Silicone Oil F/u
26 y/o M RD OS
s/p CE/PPV/MP/
retinectomy/SO

3B

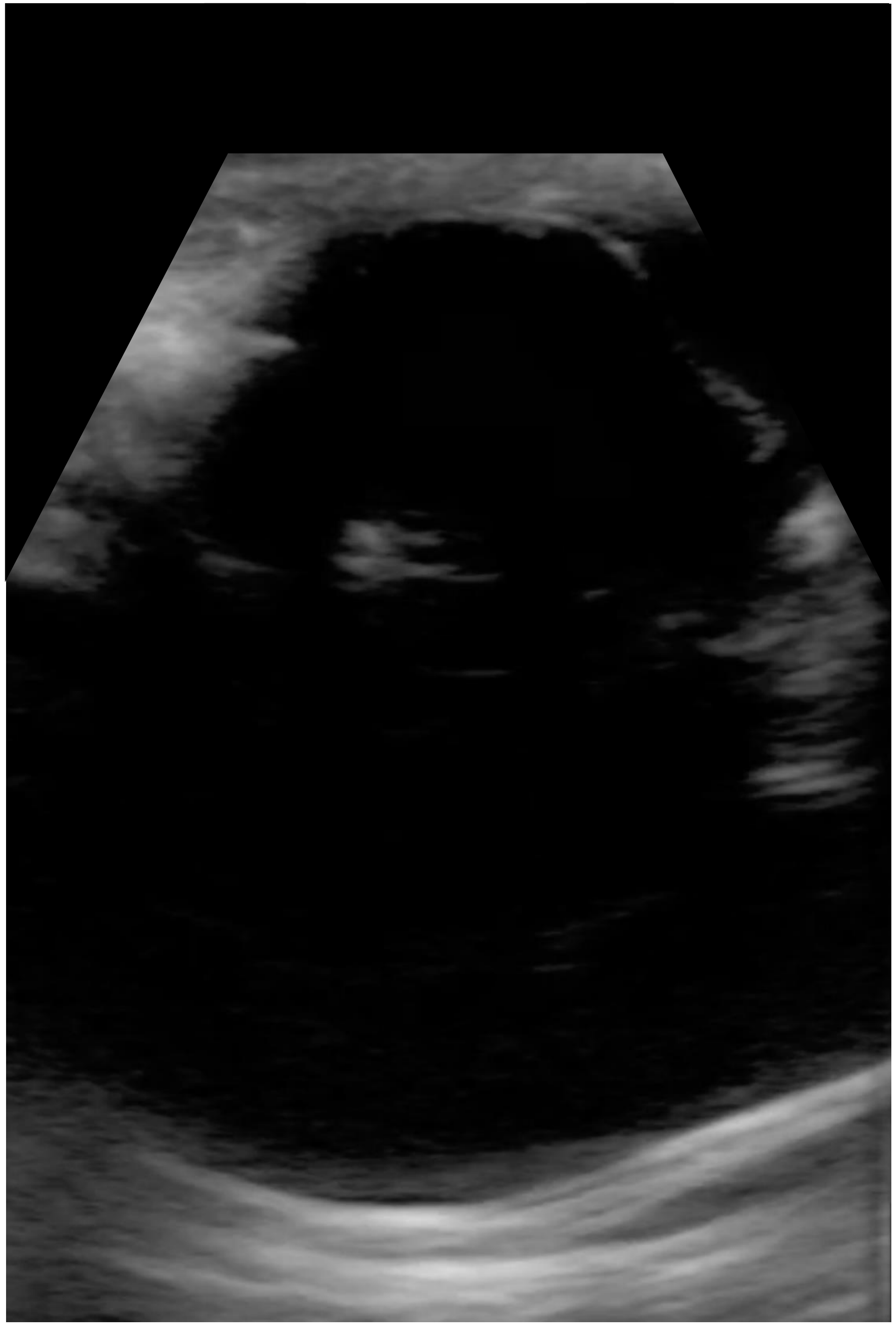

CC: Silicone Oil F/u
26 y/o M RD OS
s/p CE/PPV/MP/
retinectomy/SO

3C

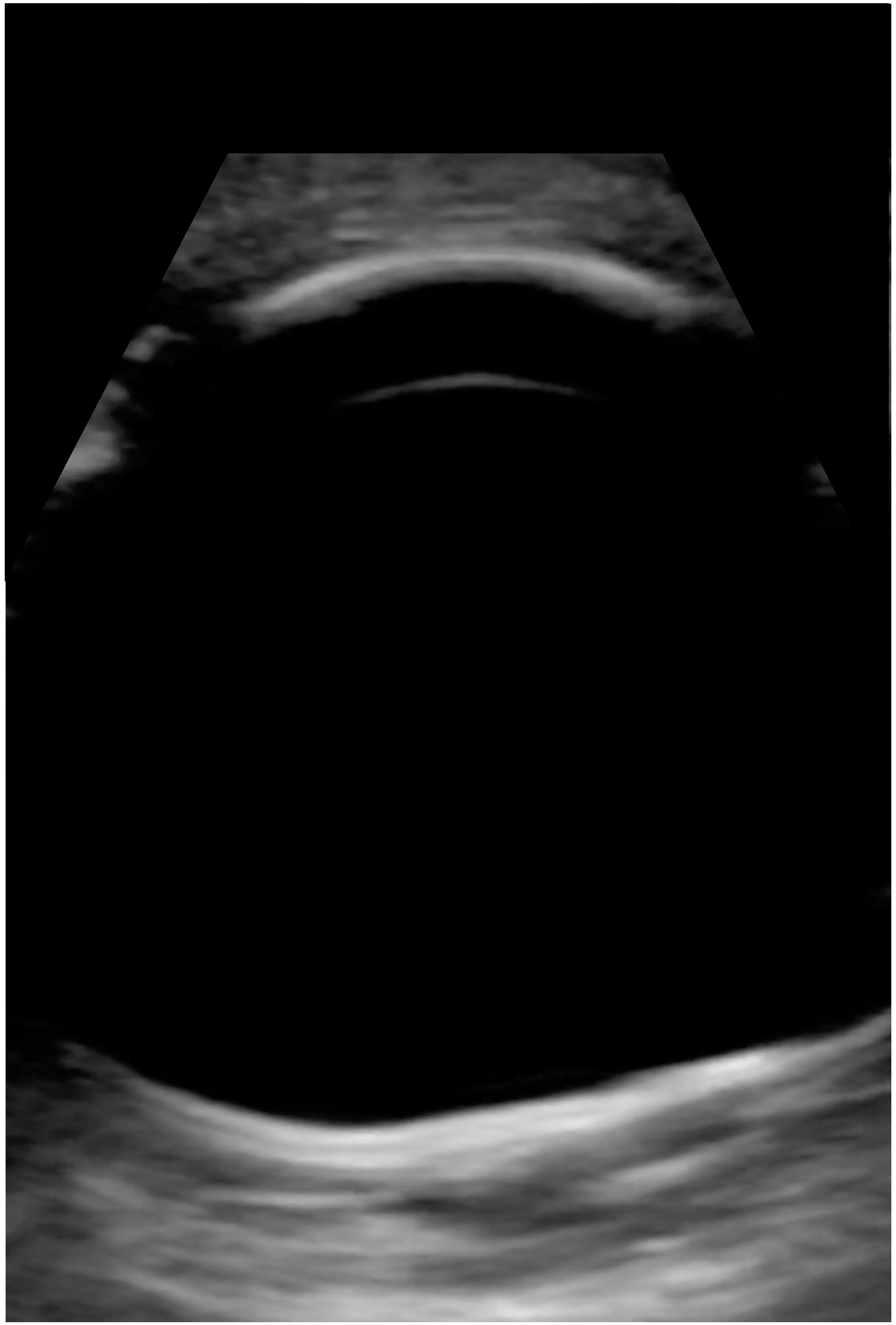

CC: Silicone Oil F/u
26 y/o M RD OS
s/p CE/PPV/MP/
retinectomy/SO

3D

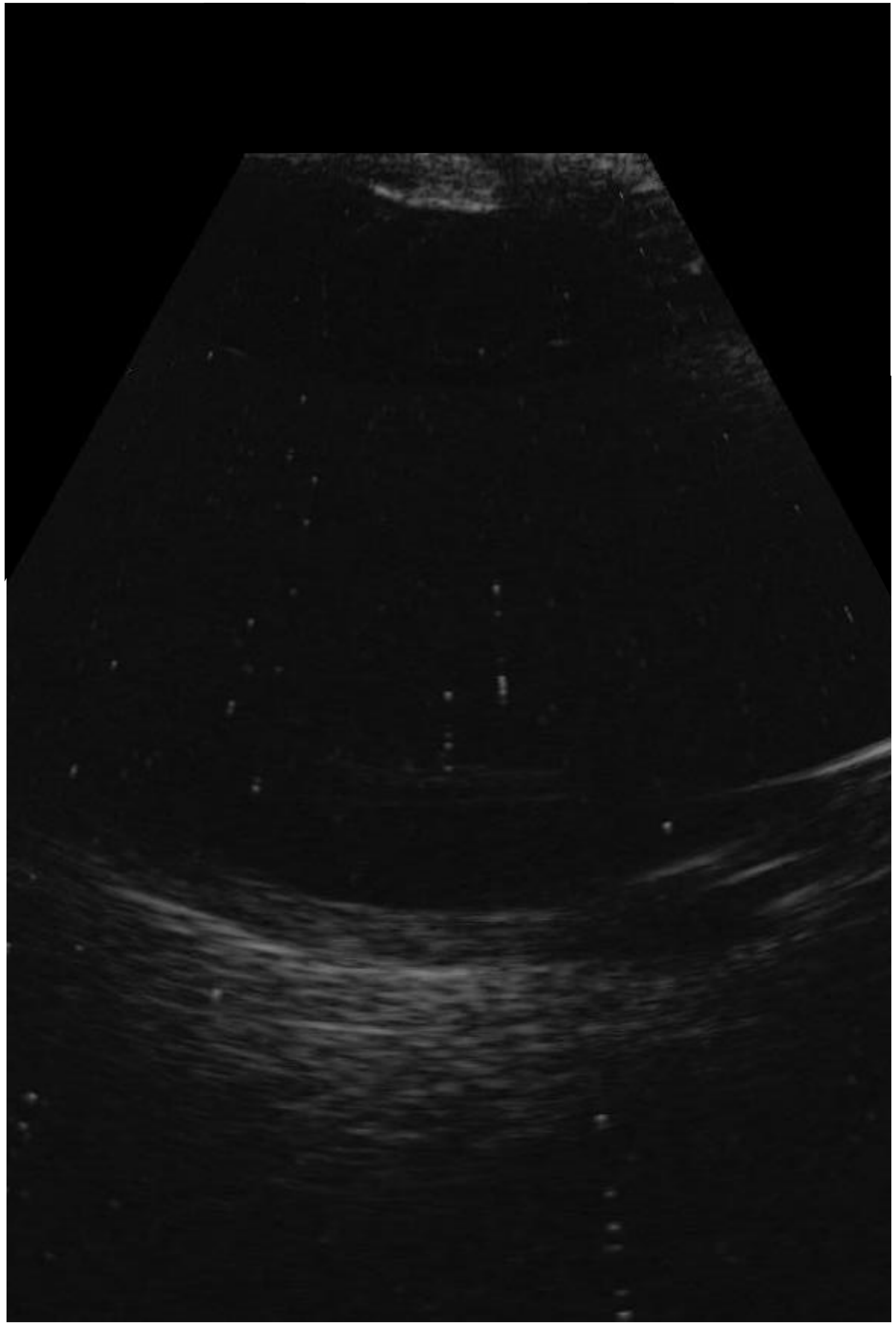

CC: Silicone Oil F/u
26 y/o M RD OS
s/p CE/PPV/MP/
retinectomy/SO

3E

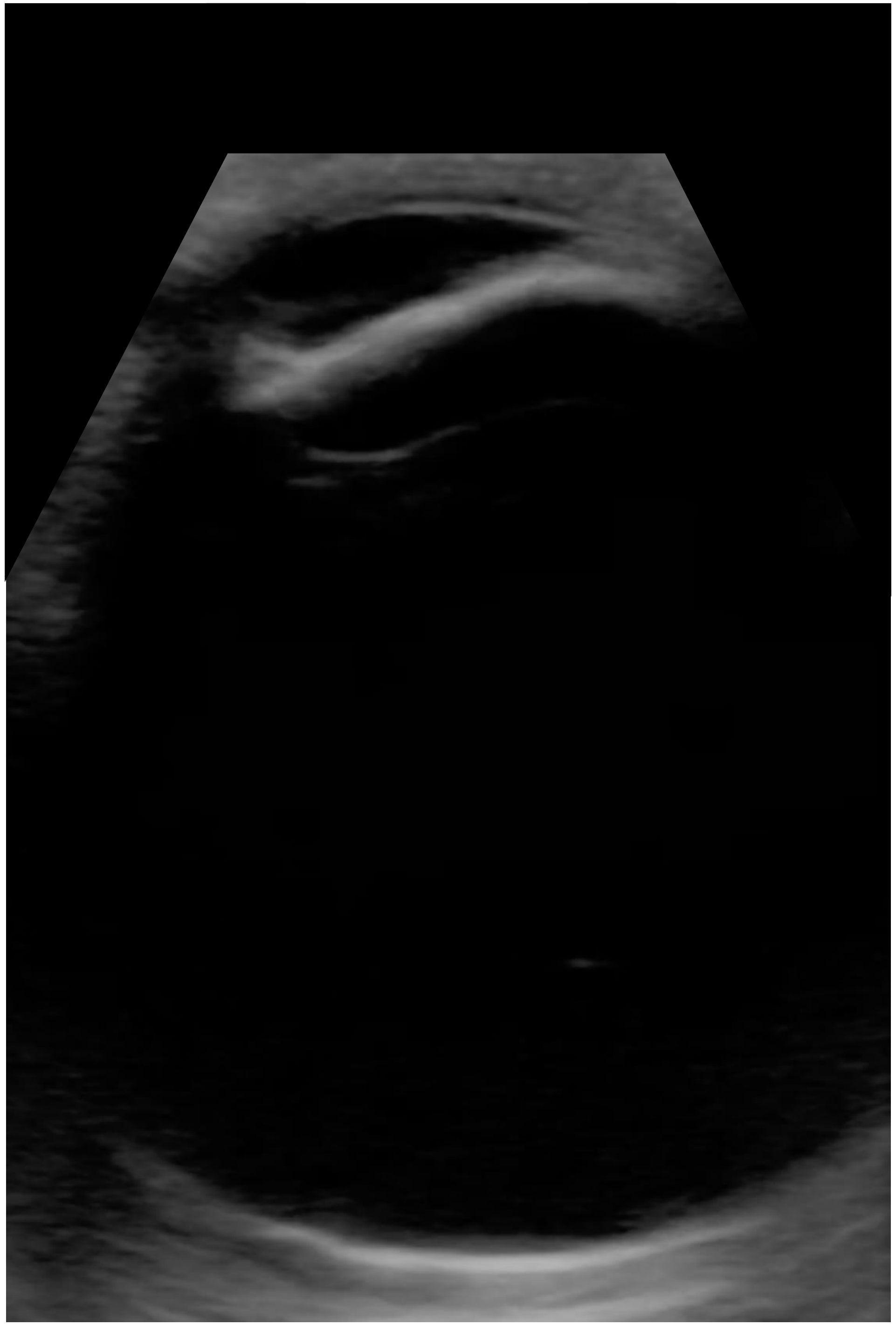

CC: Silicone Oil F/u
26 y/o M RD OS
s/p CE/PPV/MP/
retinectomy/SO

4A

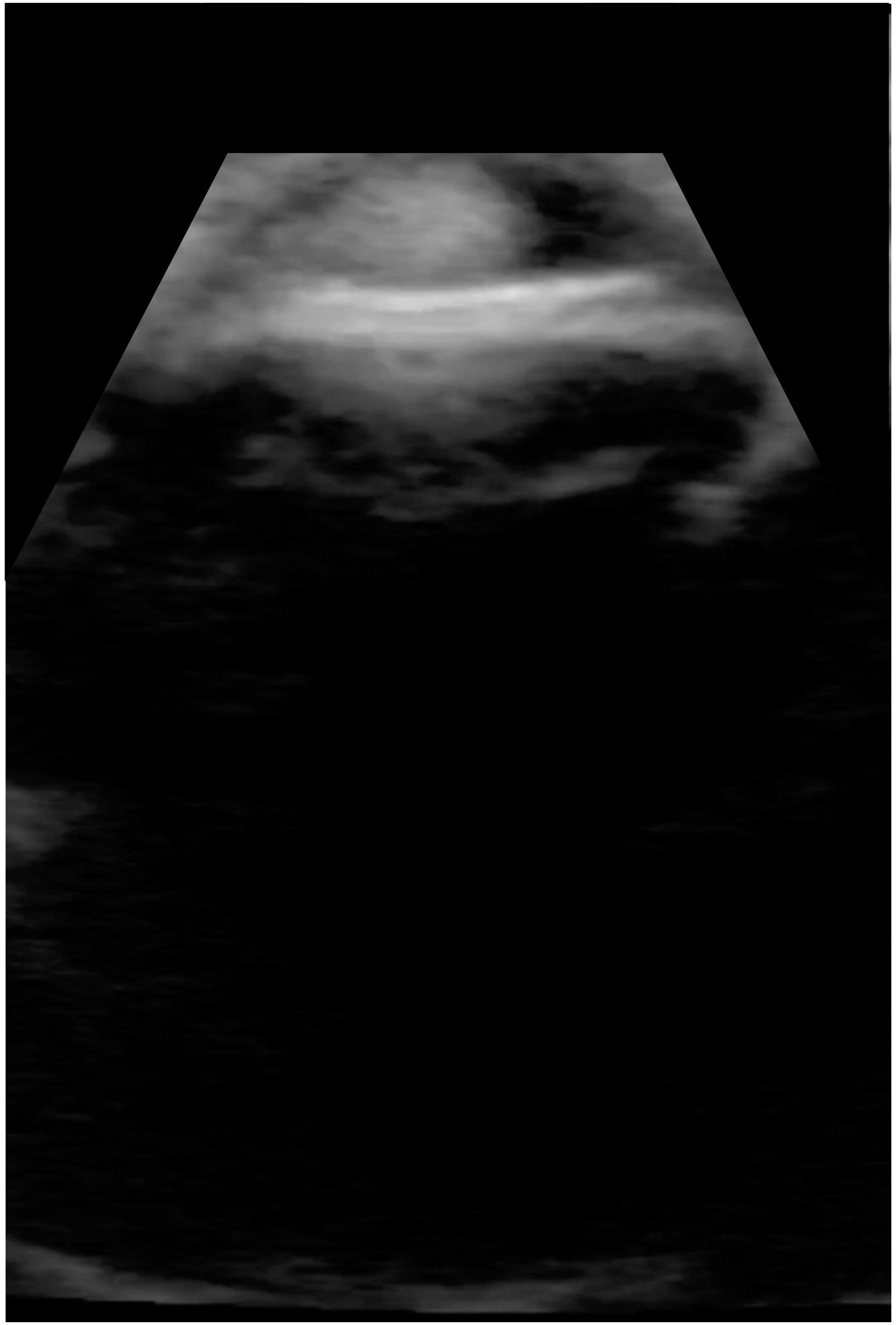

CC: Silicone Oil F/u
66 y/o M RD OD s/p PPV/SOI

4B

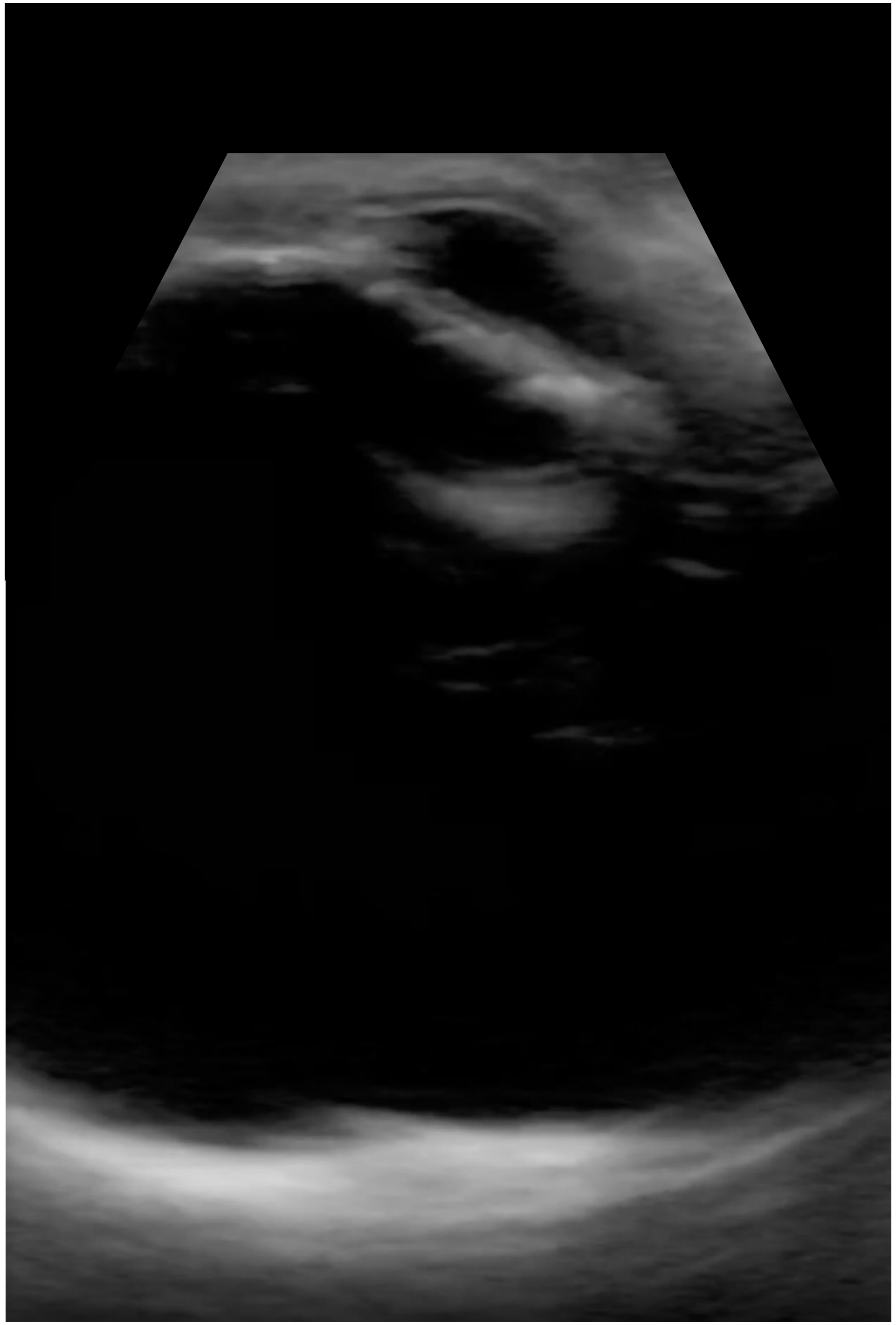

CC: Silicone Oil F/u
66 y/o M RD OD s/p
PPV/SOI

4C

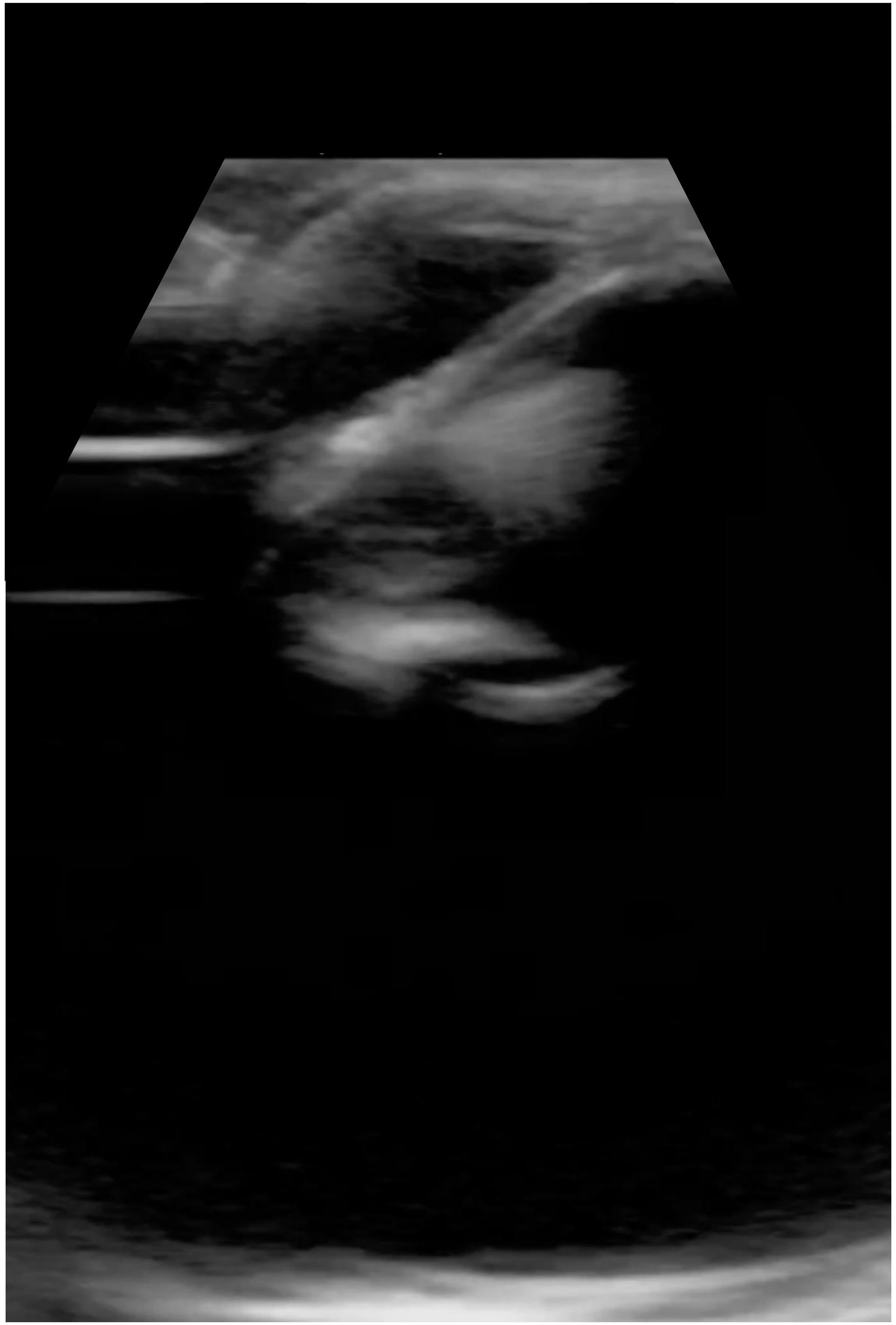

CC: Silicone Oil F/u
66 y/o M RD OD s/p
PPV/SOI

4D

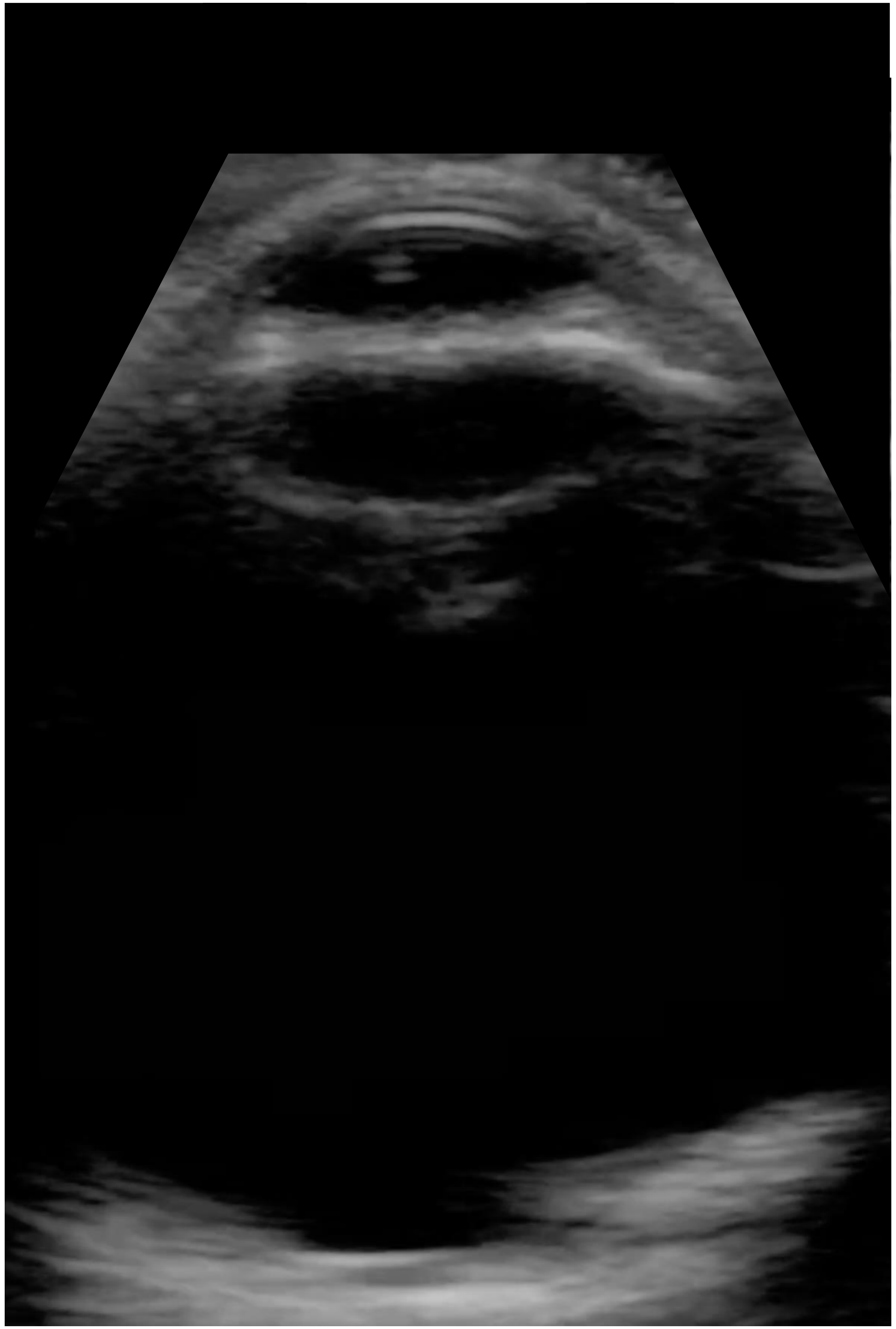

CC: Silicone Oil F/u
66 y/o M RD OD s/p PPV/SOI

4E

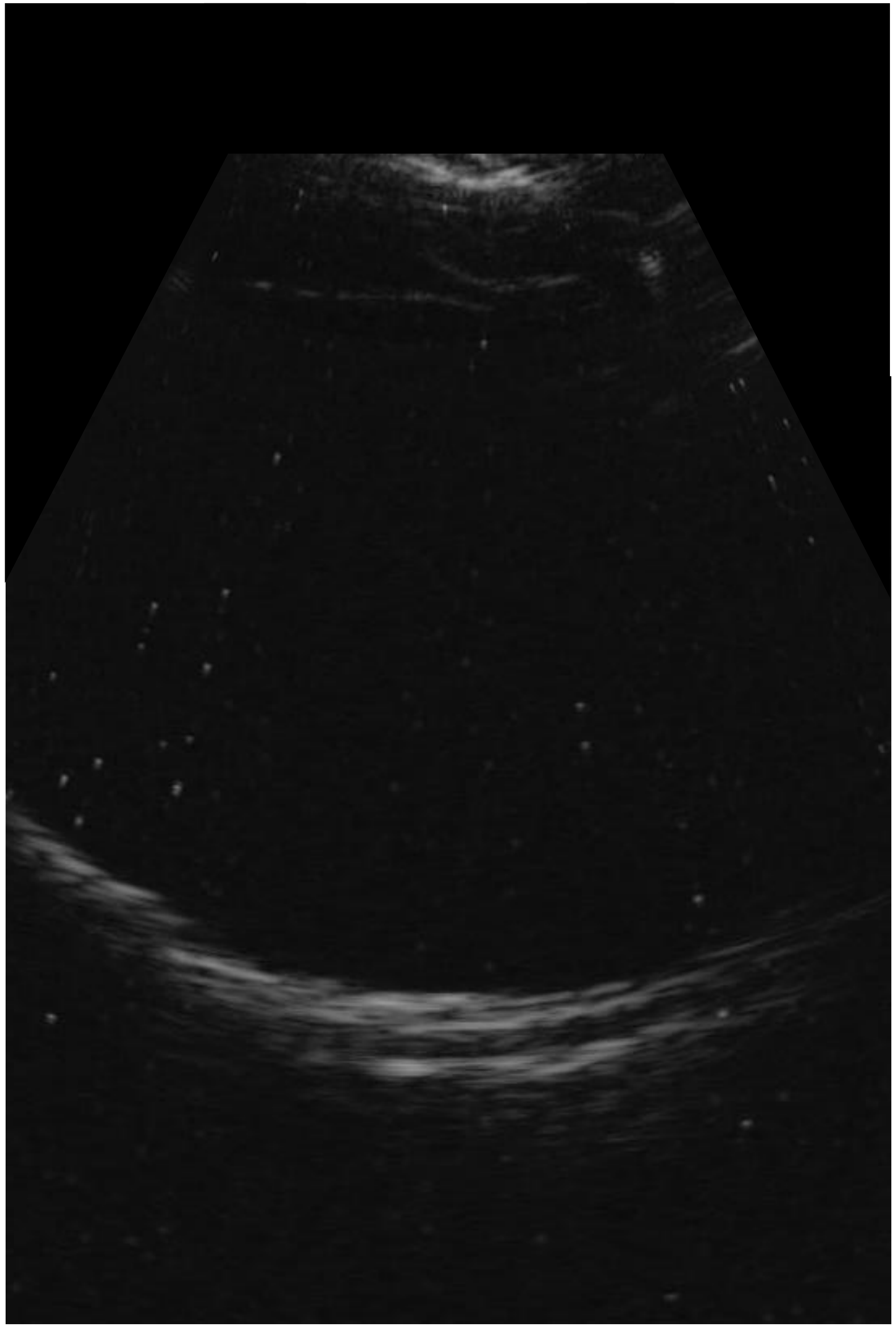

CC: Silicone Oil F/u
66 y/o M RD OD s/p PPV/SOI

5A

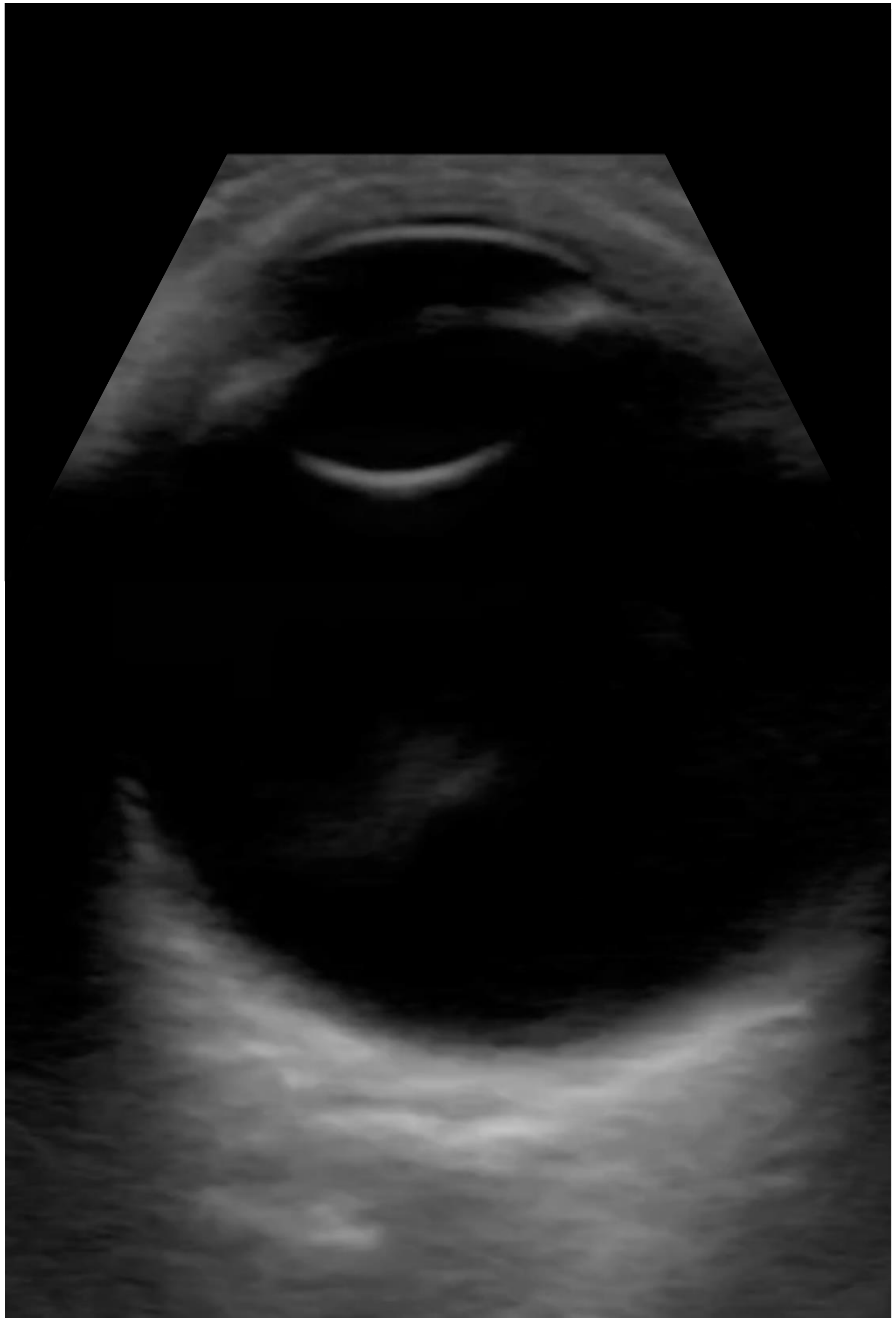

CC: CME F/u + Floaters
71y/o M w/ cystoid
macular degeneration
OD, floater w/ PPV/AFX

5B

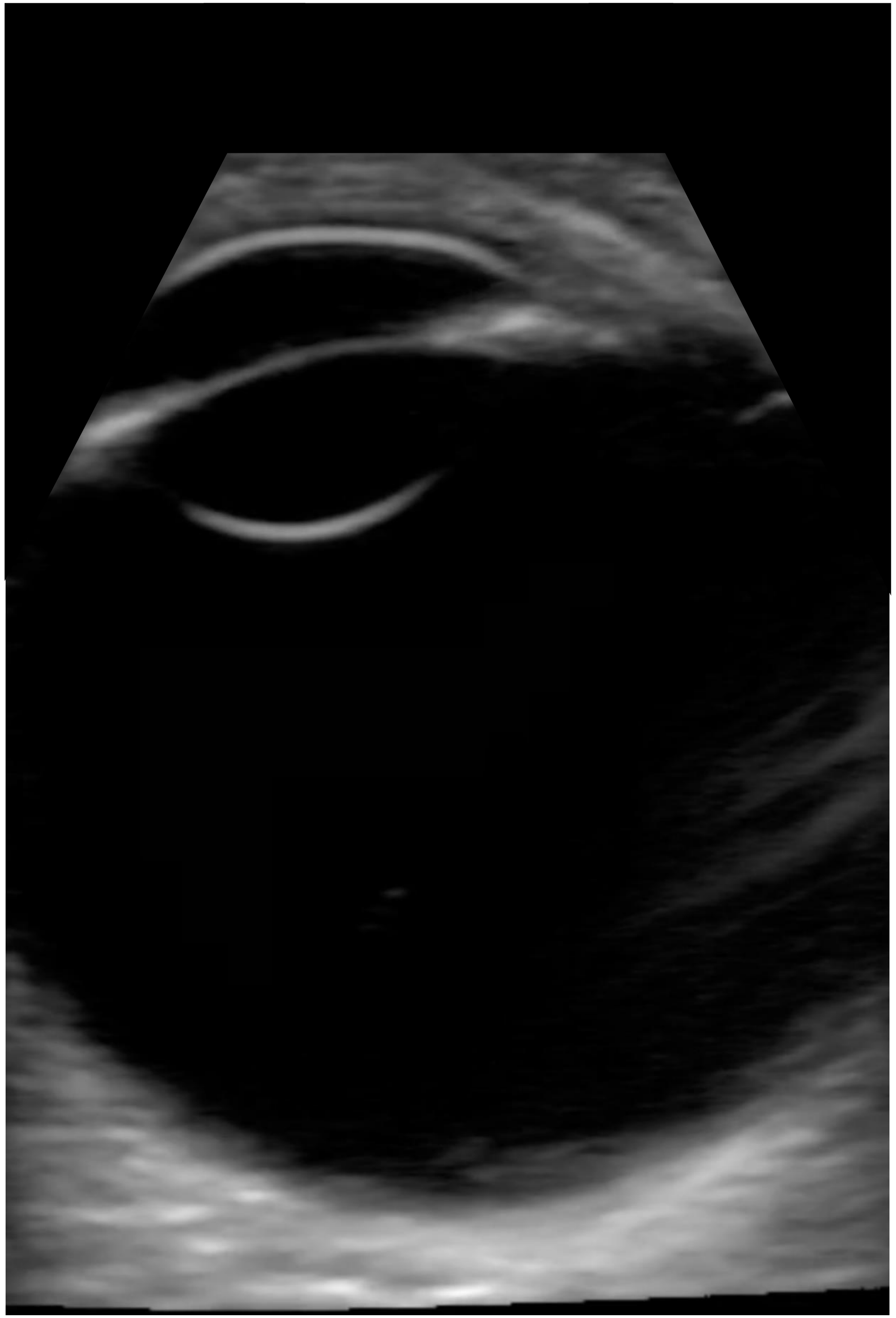

CC: CME F/u + Floaters
71y/o M w/ cystoid
macular degeneration
OD, floater w/ PPV/AFX

5C

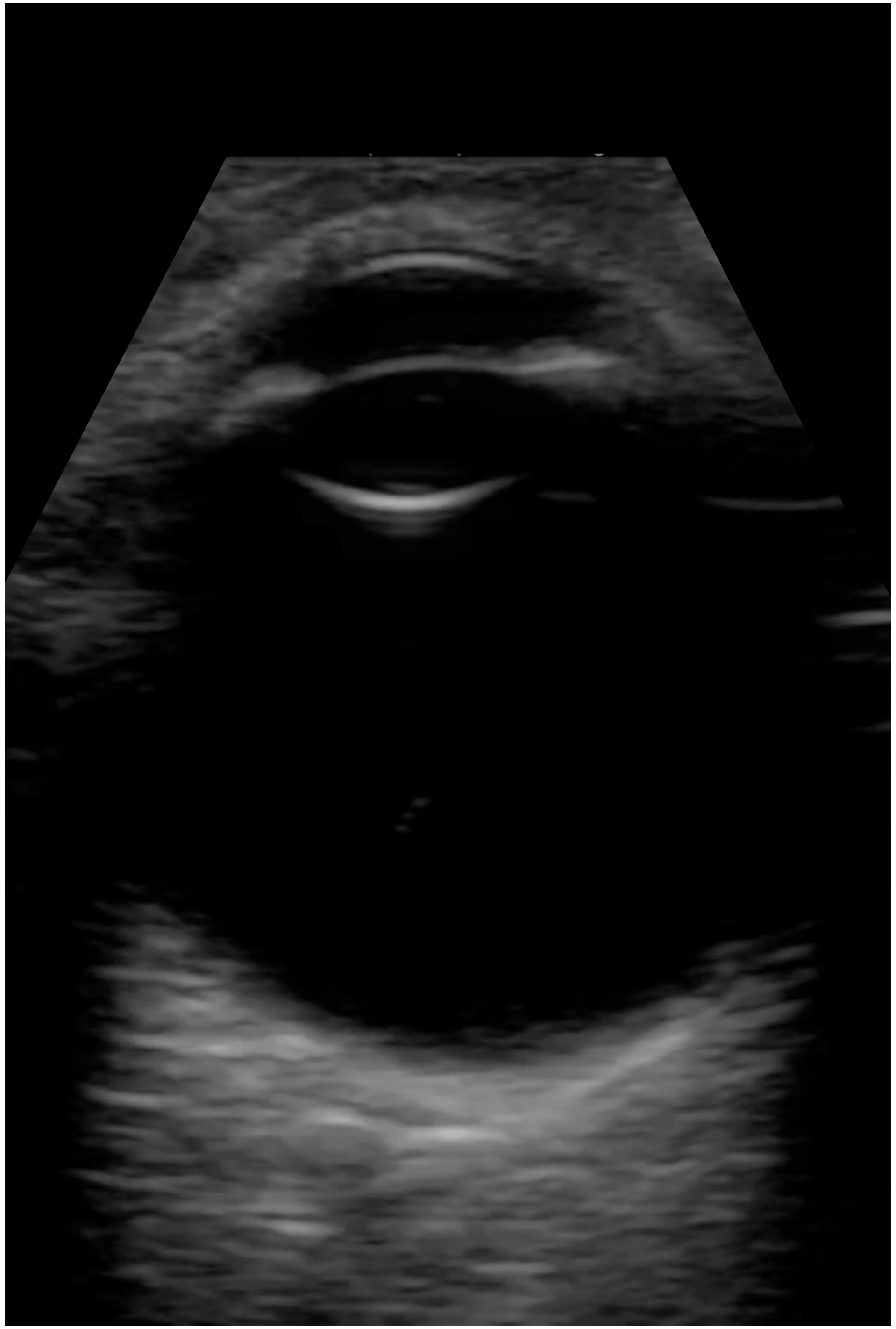

CC: CME F/u + Floaters
71y/o M w/ cystoid
macular degeneration
OD, floater w/ PPV/AFX

5D

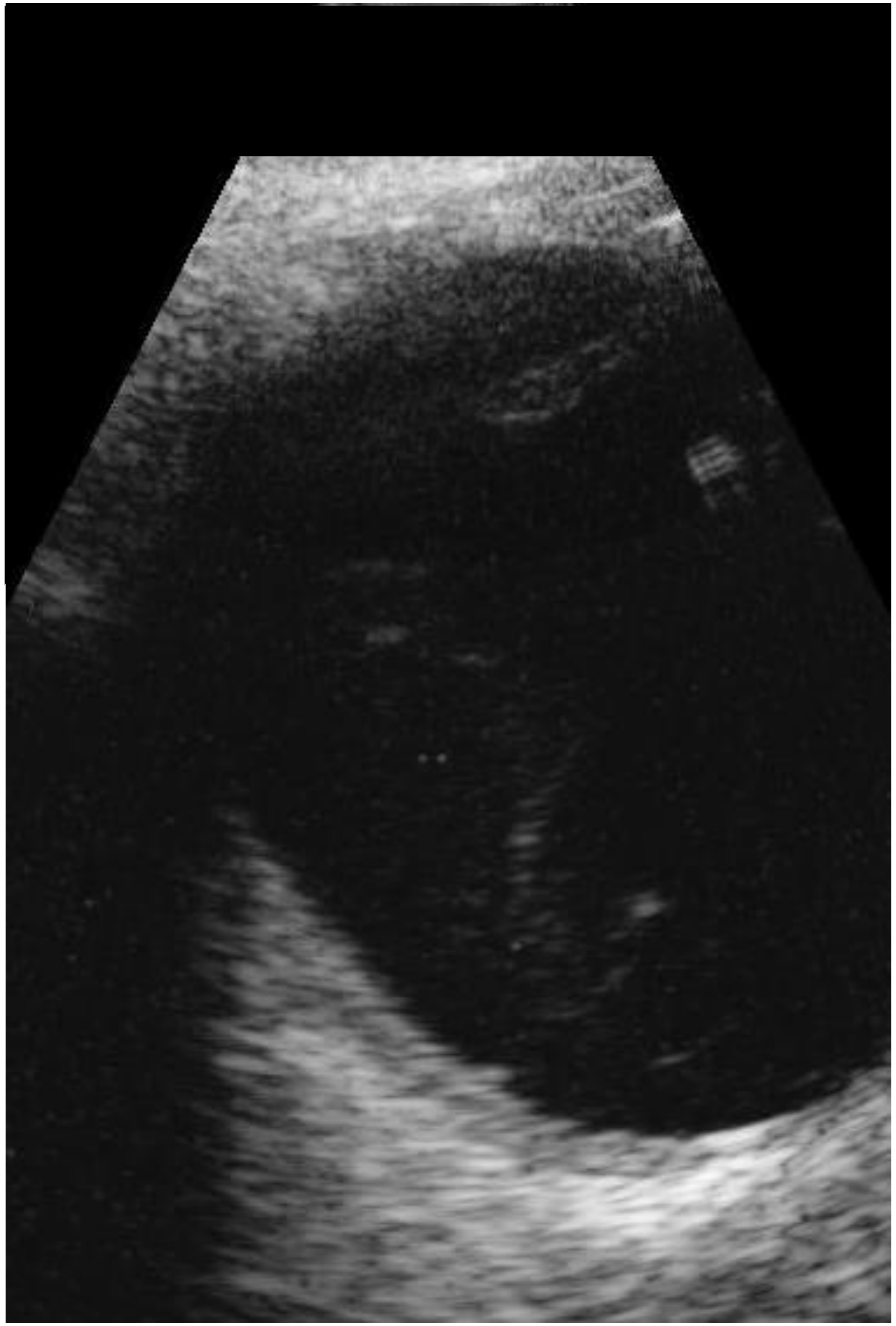

CC: CME F/u + Floaters
71y/o M w/ cystoid macular degeneration OD, floater w/ PPV/AFX

5E

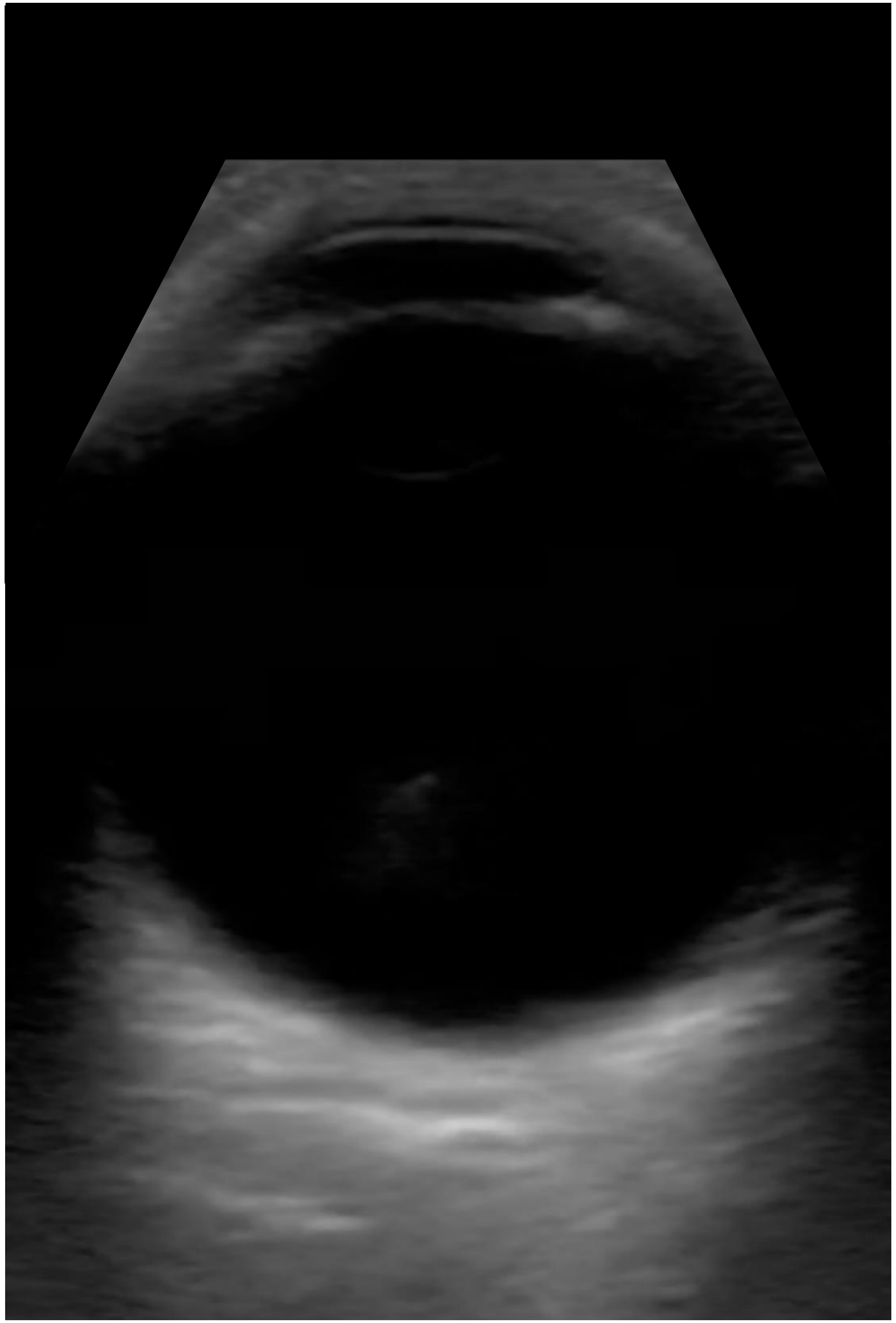

CC: CME F/u + Floaters
71y/o M w/ cystoid macular degeneration OD, floater w/ PPV/AFX

6A

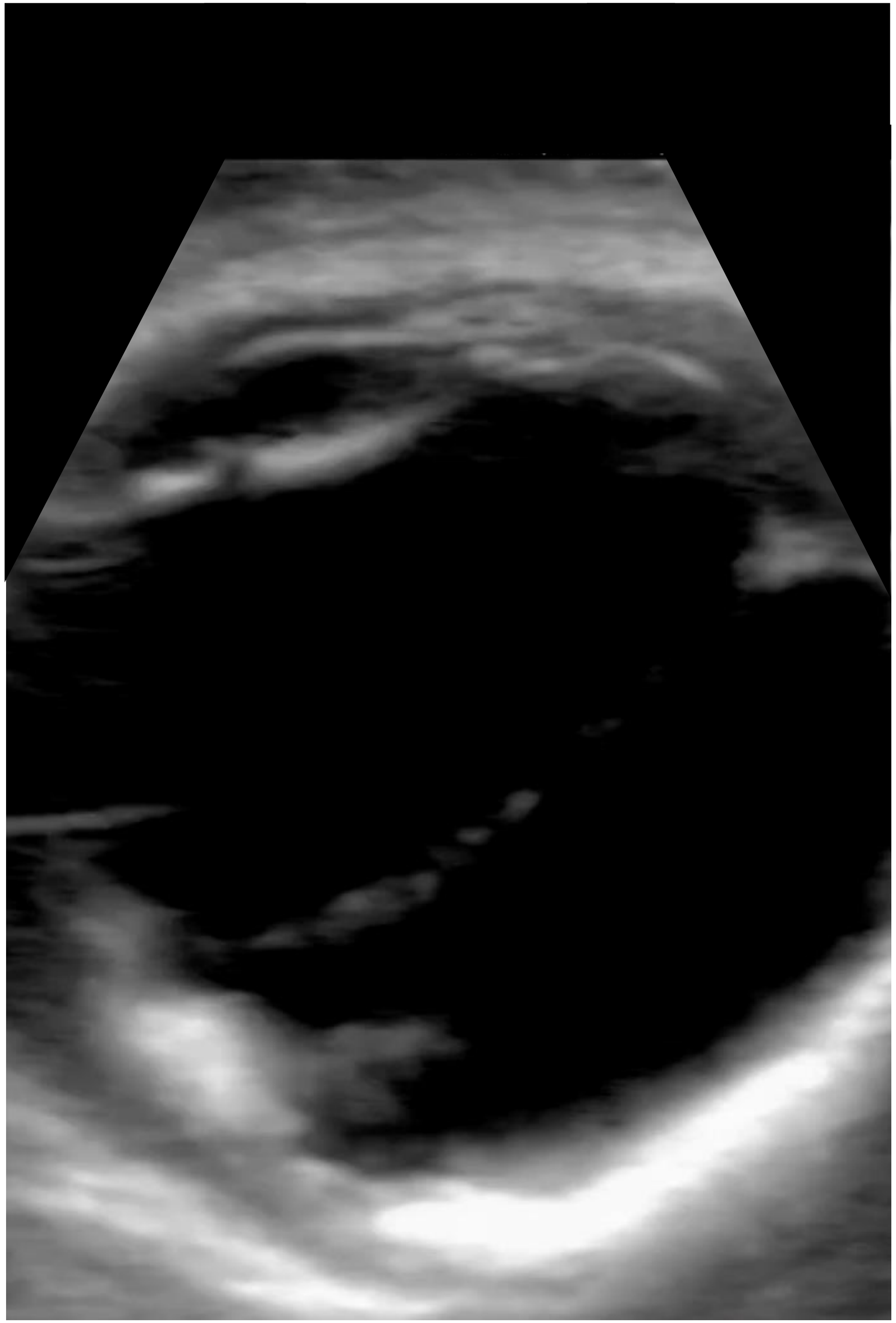

CC: OS Vit Heme F/u
66 y/o M w/ h/o TRD OS, HIV, Aspergillus, tractional band, and diffuse retinal calcifications

6B

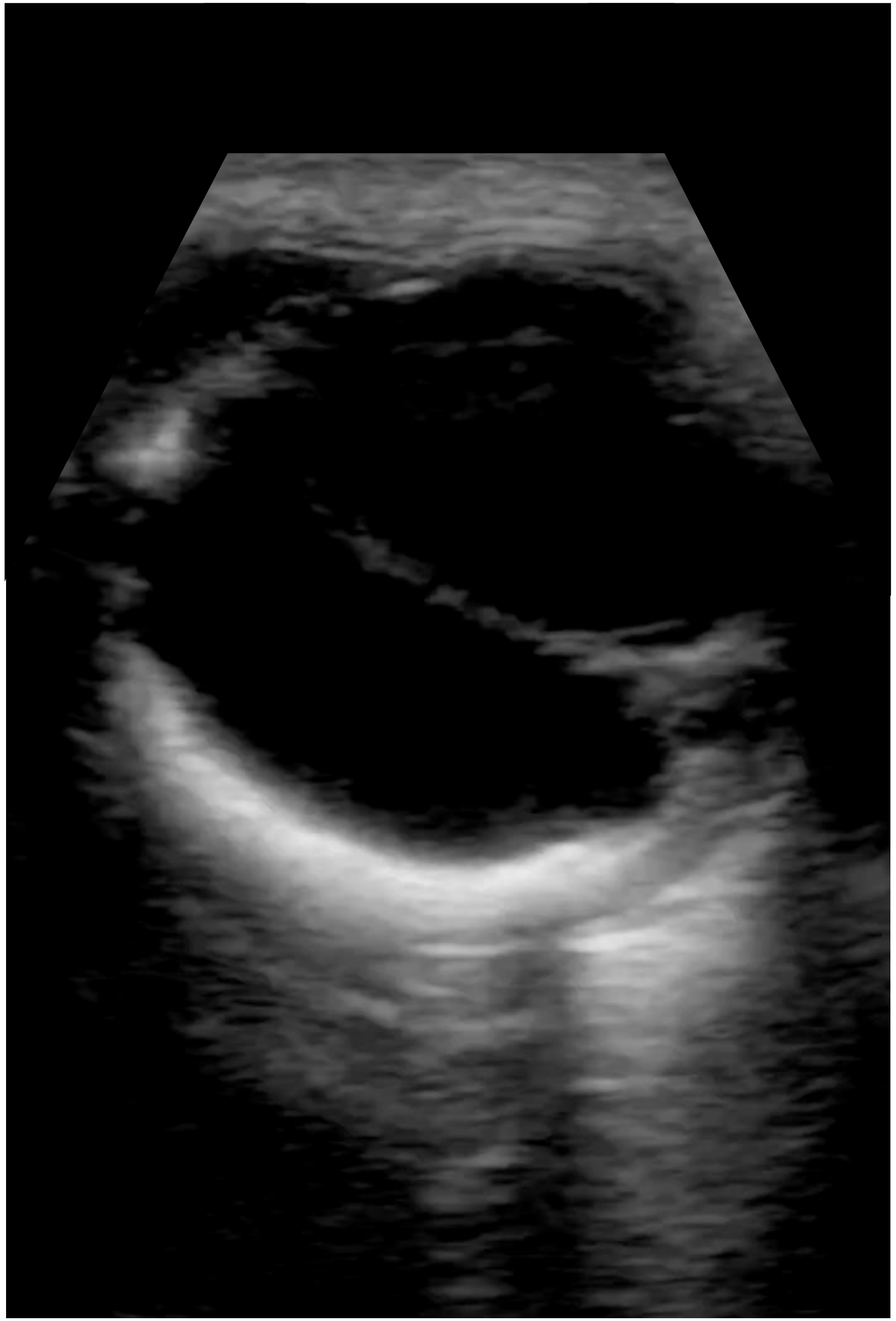

CC: OS Vit Heme F/u
66 y/o M w/ h/o TRD OS, HIV, Aspergillus, tractional band, and diffuse retinal calcifications

6C

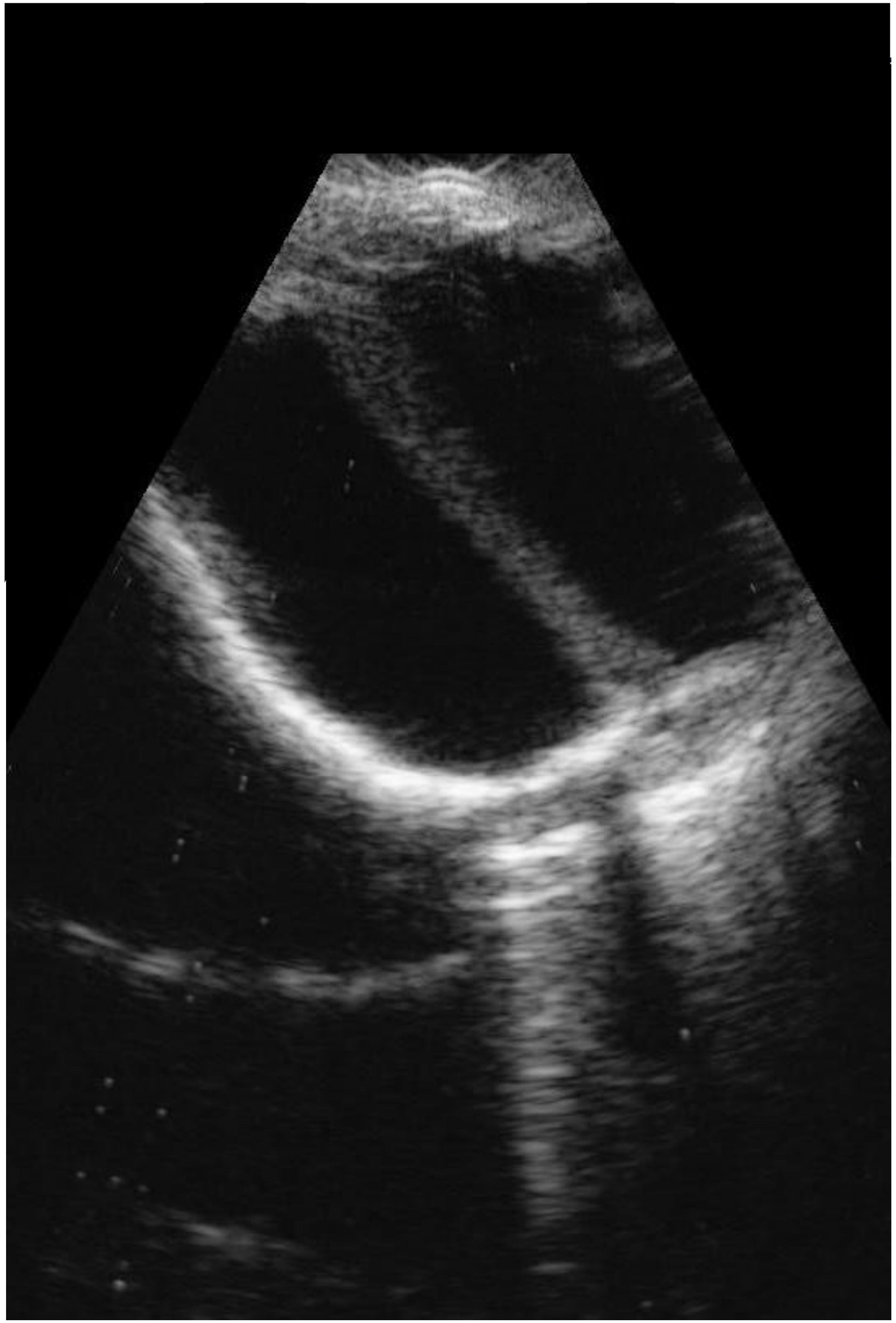

CC: OS Vit Heme F/u
66 y/o M w/ h/o TRD OS, HIV, Aspergillus, tractional band, and diffuse retinal calcifications

6D

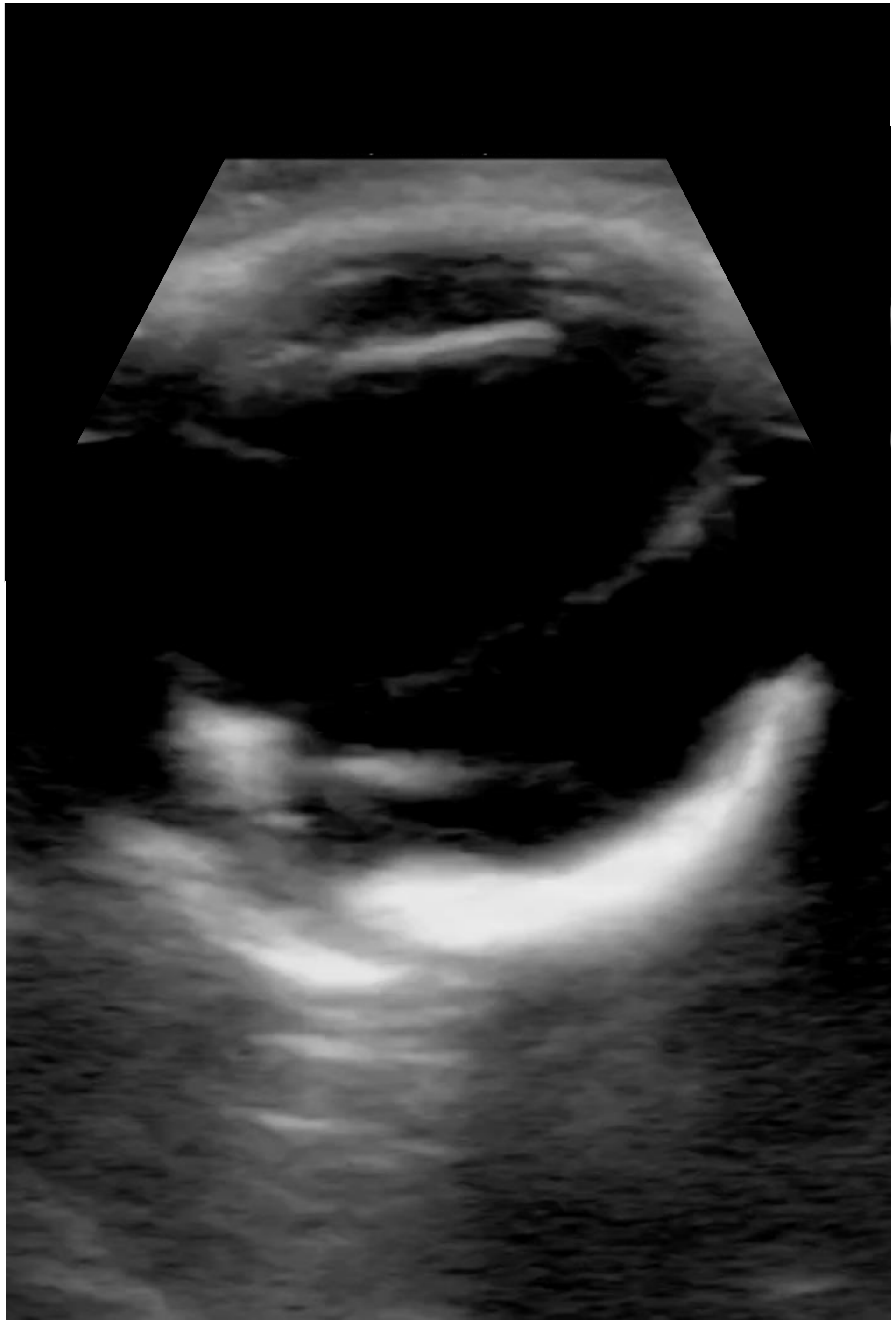

CC: OS Vit Heme F/u
66 y/o M w/ h/o TRD OS, HIV, Aspergillus, tractional band, and diffuse retinal calcifications

6E

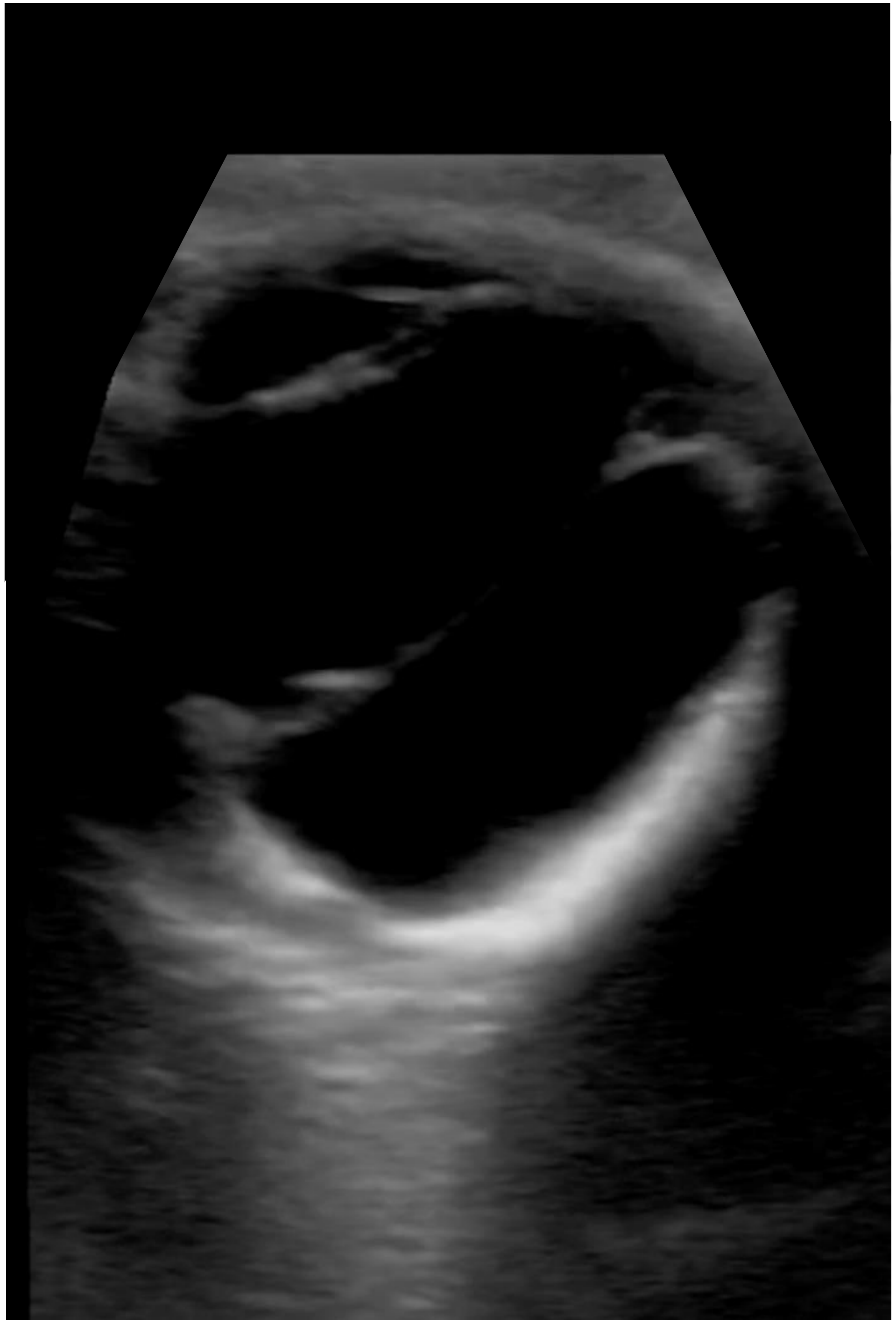

CC: OS Vit Heme F/u
66 y/o M w/ h/o TRD OS, HIV, Aspergillus, tractional band, and diffuse retinal calcifications

7A

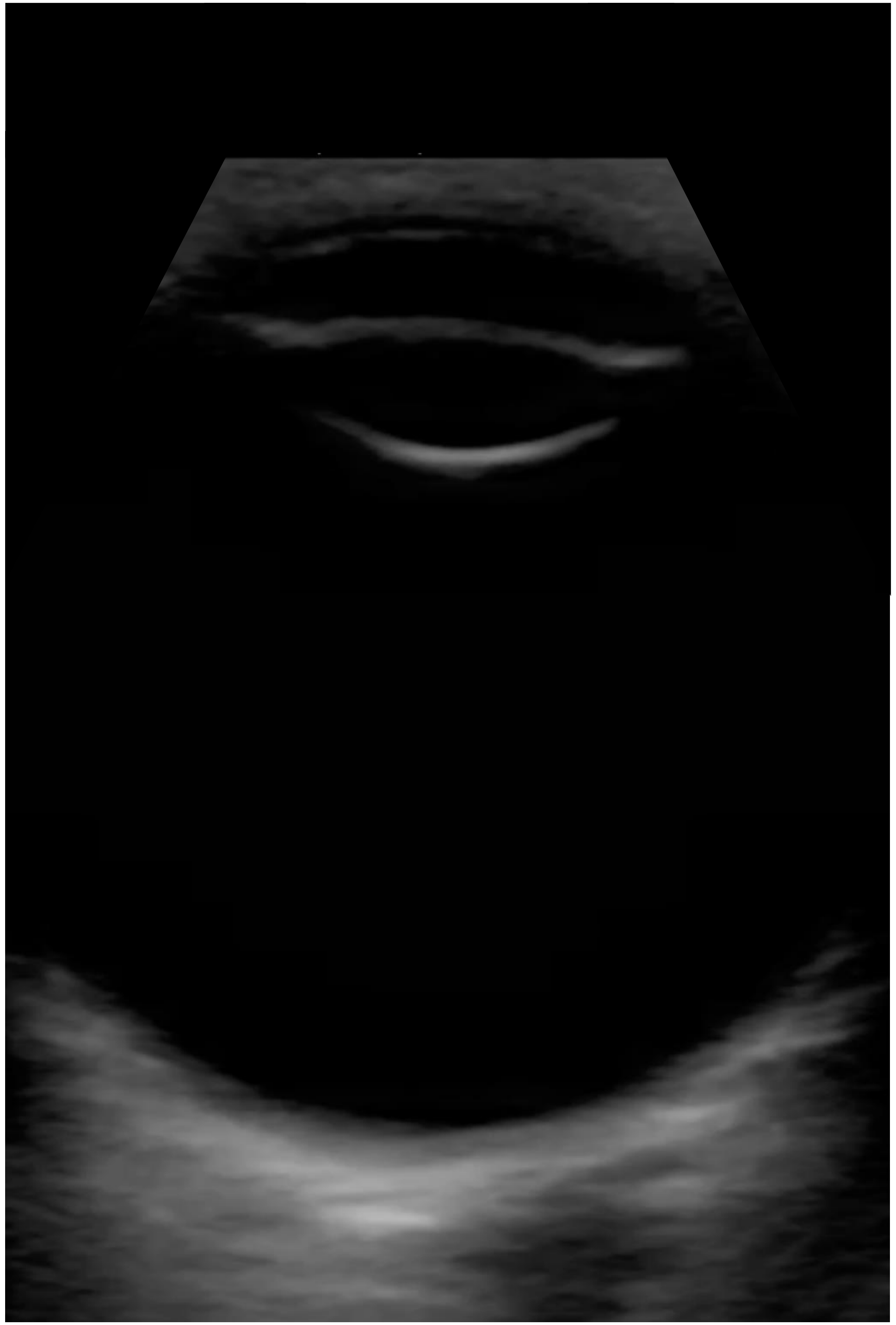

CC: Routine Visit
Healthy Subject no POH

7B

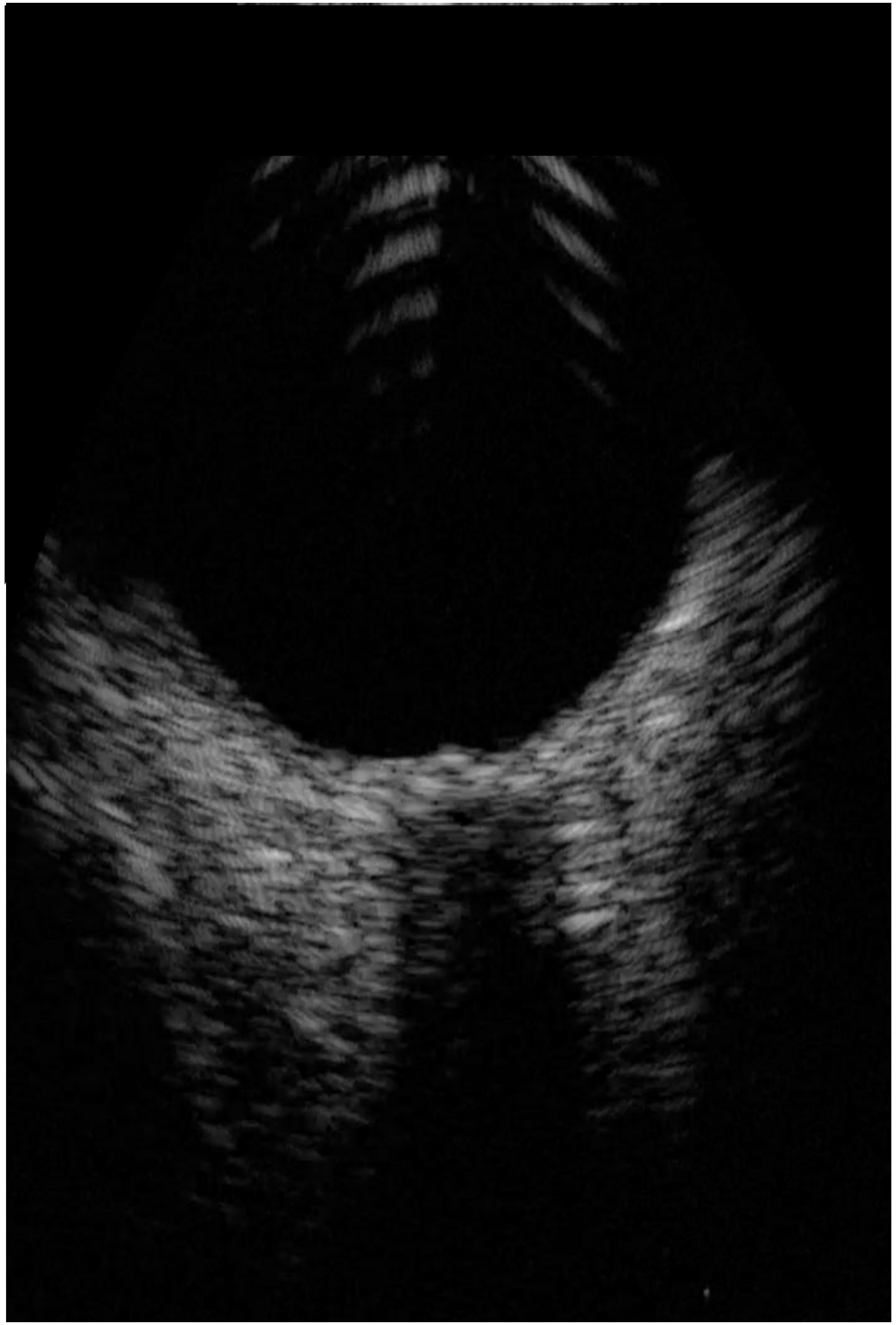

CC: Routine Visit
Healthy Subject no POH

7C

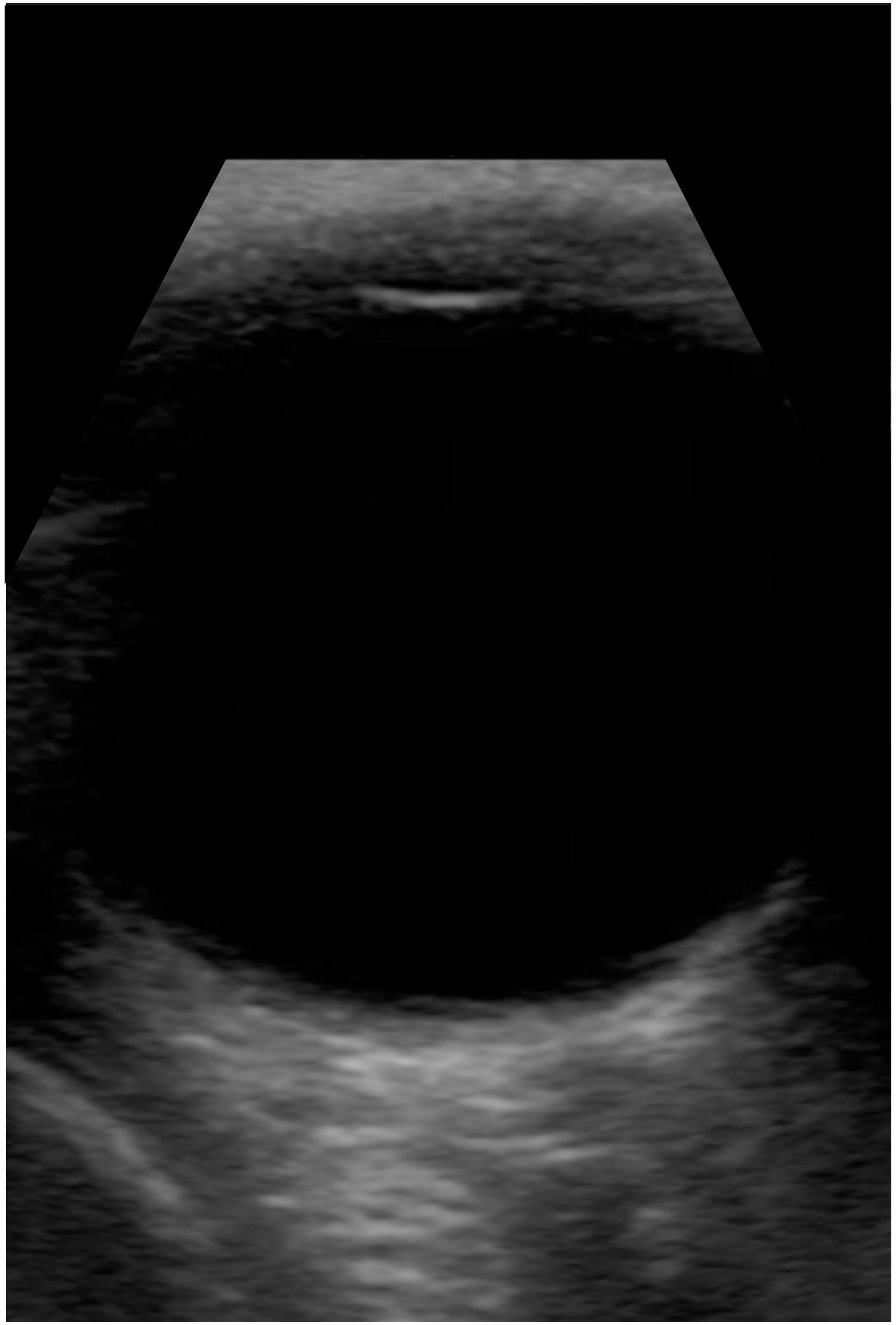

CC: Routine Visit
Healthy Subject no POH

7D

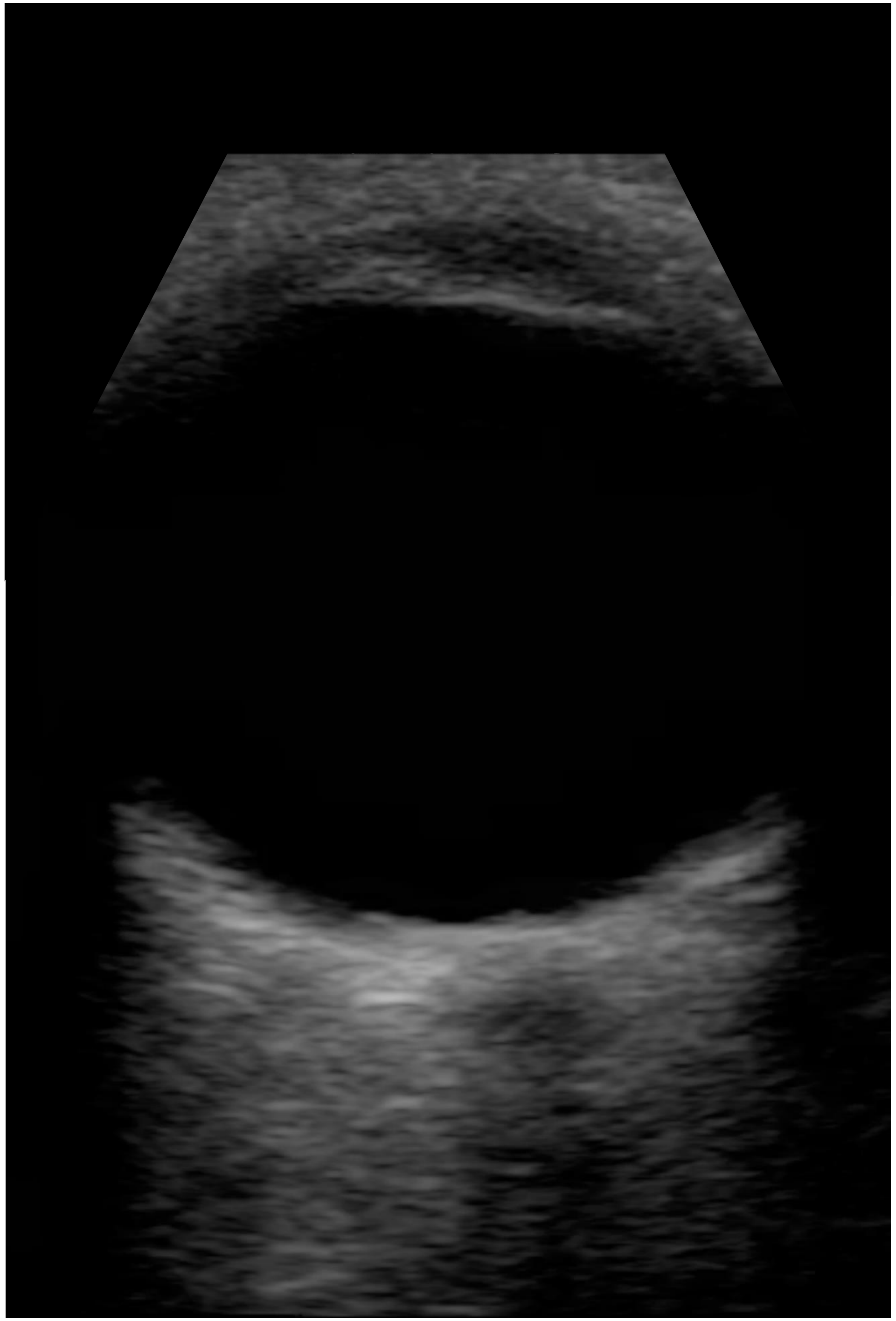

CC: Routine Visit
Healthy Subject no POH

7E

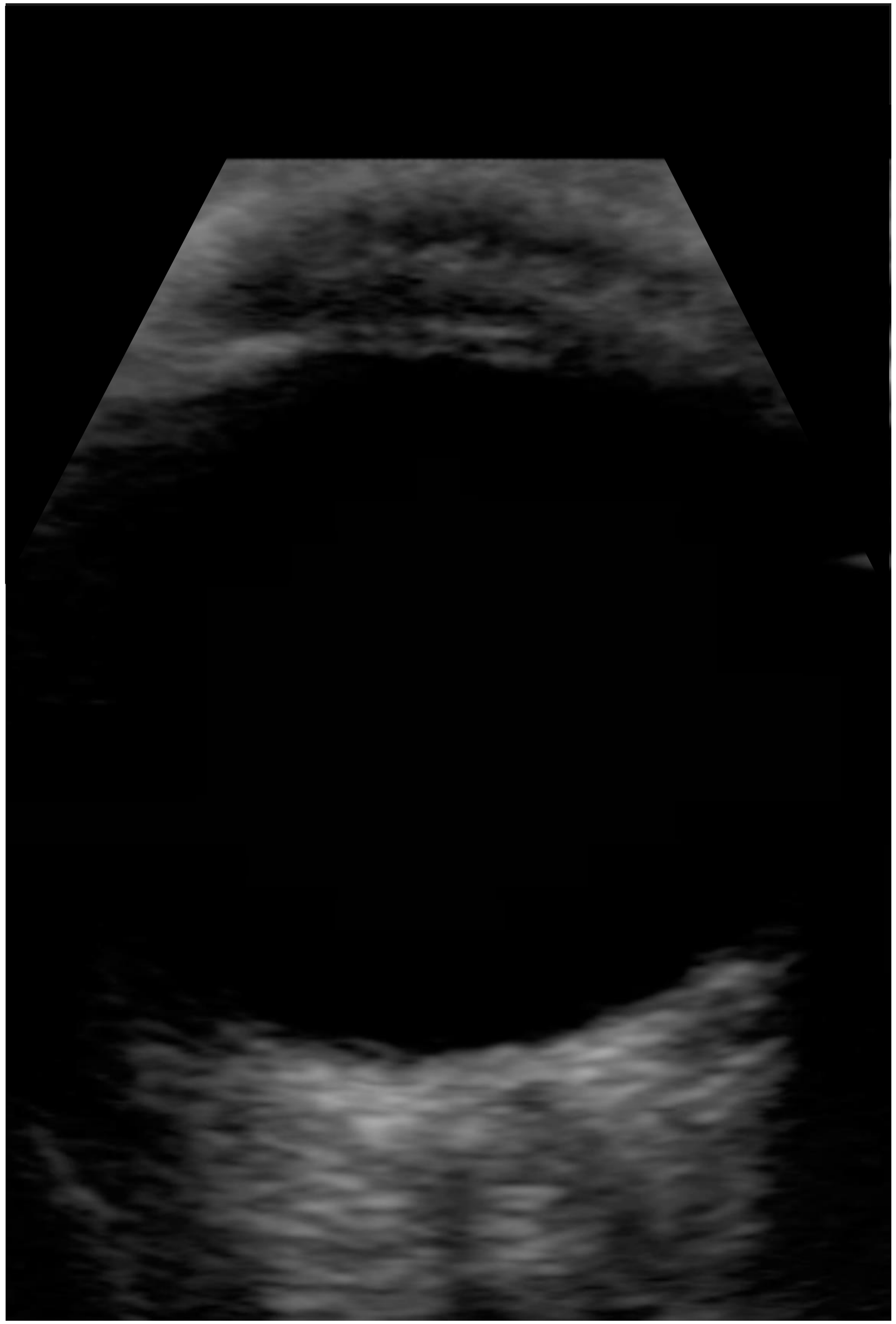

CC: Routine Visit
Healthy Subject no POH
