# Supplementary material for: Imaging performance of portable and conventional ultrasound imaging technologies for ophthalmic applications
Source: PLoS One. 2024 May 13;19(5):e0300451. doi: 10.1371/journal.pone.0300451 (PMC11090327; doi:10.1371/journal.pone.0300451)
Supplement: Attachment — Submitted filename: Response to Reviewers.docx [file pone.0300451.s006.docx]

**Response to Reviewers**

We are enclosing a revised version of our manuscript entitled “Imaging performance of portable and conventional ultrasound imaging technologies for ophthalmic applications”. The reviewers’ comments are in blue text. Author responses are in black text.

We would like to thank the reviewers for their comments and efforts in improving our manuscript. Our revised manuscript addresses all comments made by the reviewers. A detailed, point-by-point reply to each of the comments is listed below.

Journal Requirements:

1. When submitting your revision, we need you to address these additional requirements. Please ensure that your manuscript meets PLOS ONE's style requirements, including those for file naming. The PLOS ONE style templates can be found at https://journals.plos.org/plosone/s/file?id=wjVg/PLOSOne_formatting_sample_main_body.pdf and https://journals.plos.org/plosone/s/file?id=ba62/PLOSOne_formatting_sample_title_authors_affiliations.pdf

We thank the academic editor for the above comment. We thoroughly edited the manuscript to meet PLOS ONE’s style requirements and ensured the uploaded files met requirements for file naming. The provided links to the PLOS ONE templates were used to guide formatting and submission requirements at https://journals.plos.org/plosone/s/submission-guidelines were fully reviewed.

2. We suggest you thoroughly copyedit your manuscript for language usage, spelling, and grammar. If you do not know anyone who can help you do this, you may wish to consider employing a professional scientific editing service.

Whilst you may use any professional scientific editing service of your choice, PLOS has partnered with both American Journal Experts (AJE) and Editage to provide discounted services to PLOS authors. Both organizations have experience helping authors meet PLOS guidelines and can provide language editing, translation, manuscript formatting, and figure formatting to ensure your manuscript meets our submission guidelines. To take advantage of our partnership with AJE, visit the AJE website (http://aje.com/go/plos) for a 15% discount off AJE services. To take advantage of our partnership with Editage, visit the Editage website (www.editage.com) and enter referral code PLOSEDIT for a 15% discount off Editage services. If the PLOS editorial team finds any language issues in text that either AJE or Editage has edited, the service provider will re-edit the text for free. Upon resubmission, please provide the following:

The name of the colleague or the details of the professional service that edited your manuscript

A copy of your manuscript showing your changes by either highlighting them or using track changes (uploaded as a *supporting information* file)

A clean copy of the edited manuscript (uploaded as the new *manuscript* file).

We would like to thank the editor for their feedback and recommendation of employing a copyeditor to review the manuscript for language usage, spelling, and grammar. Our manuscript was reviewed and edited by our colleague Angele Nalbandian, PhD. We have uploaded a copy of our manuscript with tracked changes that reflects our copyeditor’s edits and a clean copy of the manuscript.

3. In your Data Availability statement, you have not specified where the minimal data set underlying the results described in your manuscript can be found. PLOS defines a study's minimal data set as the underlying data used to reach the conclusions drawn in the manuscript and any additional data required to replicate the reported study findings in their entirety. All PLOS journals require that the minimal data set be made fully available. For more information about our data policy, please see <http://journals.plos.org/plosone/s/data-availability>.

Upon re-submitting your revised manuscript, please upload your study’s minimal underlying data set as either Supporting Information files or to a stable, public repository and include the relevant URLs, DOIs, or accession numbers within your revised cover letter. For a list of acceptable repositories, please see http://journals.plos.org/plosone/s/data-availability#loc-recommended-repositories. Any potentially identifying patient information must be fully anonymized.

Important: If there are ethical or legal restrictions to sharing your data publicly, please explain these restrictions in detail. Please see our guidelines for more information on what we consider unacceptable restrictions to publicly sharing data: http://journals.plos.org/plosone/s/data-availability#loc-unacceptable-data-access-restrictions. Note that it is not acceptable for the authors to be the sole named individuals responsible for ensuring data access.

We will update your Data Availability statement to reflect the information you provide in your cover letter.

We would like to thank the journal for the above comment. Our study contained a survey of three ophthalmologists comparing clinical images from the Butterfly iQ and conventional ophthalmic ultrasound. The dataset underlying the results of this survey was not provided in the original submission. We have uploaded the survey data in a supporting information file.

Review comments to the Author:

1. I suggest changing the terminology to focus more specifically on the Butterfly iQ itself rather than CMUT in general, since that is the only one tested, and the only one currently approved for ocular use.

We thank the reviewer for the above comment and agree with the suggestion. As the Butterfly iQ is the only CMUT tested in the study, and the only one currently approved for ocular use, the terminology in the abstract, discussion, and conclusion was changed to focus specifically on the Butterfly iQ rather than CMUT in general. CMUT is mentioned to explain the difference in technology between the Butterfly iQ and conventional ophthalmic ultrasound and to suggest future research directions, but the manuscript is otherwise focused on the Butterfly iQ specifically.

2. Line 44 – take out the second “technology”, perhaps “increased portability with decreased equipment cost”

We thank the reviewer for this feedback and helping us improve our manuscript. We removed the second “technology” and rephrased the sentence to “Recent advancements in ultrasound technology offer increased portability with decreased equipment costs, potentially expanding access to ultrasound imaging.”

3. Reconsider the use ofthe abbreviation BiQ. Have never seen that used.

Upon reconsideration, we agree with the reviewer and have removed the abbreviation BiQ as it is not commonplace in the literature.

4. Move the sentence starting on line 63 “These… 2D gray-scale image” to the second sentence of the same paragraph because all ultrasound waves, regardless of generating technique, function this way. Then contrast how the waves are produced.

We agree with the reviewer that the sentence starting on line 63 is better placed as the second sentence of the same paragraph. The sentence was also rephrased for clarity.

5. The authors went into great detail describing the making of the phantoms, but then didn’t really describe how they were to be systematically evaluated. Then they are discussed again in the results, but it is still unclear who did the evaluating, or by what standards.

We thank the reviewer for identifying this in our manuscript. To detail how the ultrasound images of the phantoms were systematically evaluated, we added the following to the methods section: “Ultrasound images of the phantoms were independently reviewed by the authors, comprised of a team including a retina specialist, two postdoctoral researchers, and three medical students. Phantom images were examined for identification of features designed to test for axial and lateral resolution.” The following was added to the results section to detail the result of the independent review of the phantom imaging by the authors: “The authors unanimously agreed on which phantom features were identified on imaging.”

6. The results paragraph regarding the phantoms and corresponding Figure 3 are difficult to interpret.

We thank the reviewer for identifying this section of the manuscript that could be improved for clarity. The results paragraph regarding the phantoms was completely rewritten and the explanation of these results was simplified. The caption for Figure 3 was edited for clarity. The authors hope that the improved explanation of the results and revised caption make this section easier to interpret.

7. I would also consider re-writing the conclusion section. This is a very generic conclusion that could have been made without the study. I would include statements addressing the similar imaging resolution on the custom phantoms, and the statistical agreement between 2/3 blinded reviewers

The authors agree with the reviewer that the original conclusion was generic and did not address the specific results of the study. The conclusion was rewritten and addressed results from the study, including the similar imaging resolution on the custom phantoms and the statistical agreement between 2/3 blinded reviewers.
