# Supplementary material for: Imaging performance of portable and conventional ultrasound imaging technologies for ophthalmic applications
Source: PLoS One. 2024 May 13;19(5):e0300451. doi: 10.1371/journal.pone.0300451 (PMC11090327; doi:10.1371/journal.pone.0300451)
Supplement: Attachment — Submitted filename: Response to Reviewers.docx [file pone.0300451.s007.docx]

**Response to Reviewers**

We are enclosing a revised version of our manuscript entitled “Imaging performance of portable and conventional ultrasound imaging technologies for ophthalmic applications”. The reviewers’ comments are in blue text. Author responses are in black text.

We would like to thank the reviewers for their comments and efforts in improving our manuscript. Our revised manuscript addresses all comments made by the reviewers. A detailed, point-by-point reply to each of the comments is listed below.

Review Comments to the Author:

Reviewer #1: The authors have successfully implemented the recommendations and improvements suggested in the first round of edits. The paper is improved in clarity and presentation.

We would like to thank Reviewer #1 for their suggestions and helping us improve our manuscript.

Reviewer #2:
1. The primary issue lies in the study's small sample size and limited clinical assessment. With only seven participants and three retina specialists included, the findings may not be representative to a wider population. It is suggested that the authors provide rationale for their choice of sample size.

We appreciate the input from Reviewer #2, acknowledging the limitation highlighted in their comment. Since our pilot study included only seven participants and three retina specialists, we recognize the inability to draw definitive conclusions applicable to a broader population based solely on this study. The primary aim of our research was to conduct a pilot study evaluating the suitability of ophthalmic imaging with Butterfly iQ. Despite the limited sample size, our goal was to investigate the feasibility of research procedures, identify potential issues, refine research questions, and establish initial data for the comparison between Butterfly iQ and conventional ophthalmic ultrasound. The insights gained from this pilot study are foundational to a separate but larger study evaluating 3D ultrasound methods using a range of ultrasound probe types to investigate ophthalmic structure and function. These subsequent studies could provide more robust findings, allowing for conclusions that are more widely applicable to diverse populations.

2. As for the clinal evaluation, it would be beneficial if the authors incorporate analysis using image processing software in addition to the specialists' ratings.

We appreciate the reviewer's insightful comment and valuable suggestion regarding the potential use of image processing software for quantitative comparisons between Butterfly iQ and conventional ophthalmic ultrasound images. We acknowledge that metrics such as contrast resolution, signal-to-noise ratio, and edge sharpness could enhance our understanding of the diagnostic capabilities of each device.

However, considering the limited sample size of seven participants in our pilot study, we believe that performing this type of analysis on a larger clinical dataset would be more appropriate to ensure robust and generalizable results. While we may not implement the suggested analysis in the current study due to its scale, we would appreciate recommendations on specific analysis methods that the reviewer believes are necessary to analyze ultrasound images from different ultrasound probe technologies.

3. The authors should provide the full names for the abbreviations, such as PL, MSK-ST, MSK, and N.

We thank the reviewer for bringing this to our attention. We have edited the manuscript and included the full names for the abbreviations: Musculoskeletal (MSK), Musculoskeletal-Soft Tissue (MSK-ST), Nerve (N), and Pediatric Lung (PL).
